# Disc‐Toroid Hybrid Lipid Nanoparticles for Efficient Drug Encapsulation and Subcutaneous Delivery

**DOI:** 10.1002/smll.202600052

**Published:** 2026-03-12

**Authors:** Zanelle van Niekerk, Rima Nuwayhid, Stefaniya Gaydarova, Eva Bittrich, Natalia Makarova, Susanne Boye, Christo Tzachev, Jan C. Simon, Sandra Franz, Albena Lederer

**Affiliations:** ^1^ Department of Chemistry and Polymer Science Stellenbosch University Matieland South Africa; ^2^ Leibniz‐Institut für Polymerforschung Dresden e.V. Dresden Germany; ^3^ Clinic for Orthopaedics Trauma Surgery and Plastic Surgery Leipzig University Hospital Leipzig Germany; ^4^ Faculty of Chemistry and Pharmacy Sofia University “St. Kliment Ohridski” Sofia Bulgaria; ^5^ Department of Dermatology, Venereology and Allergology, Medical Faculty University of Leipzig Leipzig Germany

**Keywords:** AF4, asymmetrical flow field flow fractionation, disc‐toroid hybrid, drug delivery, lipid nanoparticles, multidetection, SAXS

## Abstract

The development of effective drug delivery systems for subcutaneous or intradermal injection requires systems with improved bioavailability and biocompatibility. Systematic physicochemical and biological interrogation of carnauba‐wax/red‐palm‐oil lipid nanoparticles (LNPs) stabilized with d‐α‐tocopheryl‐PEG‐1000‐succinate and polysorbate‐40 shows that purposeful matrix engineering yields a robust sub‐50 nm carrier for under‐skin delivery. Cryo‐TEM and SAXS reveal hybrid morphology dominated by 30–40 nm toroidal disc‐shaped particles. Orthogonal analytics via multidetection asymmetrical flow field‐flow fractionation and WAXS confirm that loading with quinine or dihydroartemisinin leaves size and crystallinity unchanged, achieving approximately 90% encapsulation efficiencies and particle stability up to 18 months at 4°C. Formulations containing red palm oil and the dual‐surfactant corona exhibited reduced size dispersity compared with single‐component formulations. Long‐term viability assays in primary human fibroblasts and macrophages, and ex vivo cultured human skin, underscore excellent biocompatibility up to 0.024% (w/v) lipid. Fluorescein‐labeled LNPs traversed the dermis and hypodermis, while only nanomolar lipid concentrations appeared in the receiver medium, indicating a sustained local depot. Overall, this study provides insights into the relationship between formulation composition, particle morphology and measured physicochemical and biological properties relevant to under‐skin administration.

## Graphical abstract

This study demonstrates sub‐50 nm solid‐lipid nanoparticles with long‐term stability, high drug loading, and strong tissue compatibility. Disc‐toroid morphology is confirmed and may play a role in cellular interactions, while low cytotoxicity and minimal systemic permeation confirm depot‐like behavior. These nanoparticles represent a versatile platform for minimally invasive therapeutic delivery, warranting future in vivo validation.

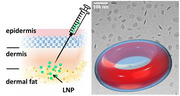

**Abbreviations**
AF4: asymmetrical flow‐field flow fractionation
MD: multidetector
MALS: multi‐angle light scattering
DLS: dynamic light scattering
SLD: scattering length density
SAXS: small‐angle X‐ray scattering
WAXS: wide‐angle X‐ray scattering
CCR2: CC chemokine receptor 2
CCL2: CC chemokine ligand 2
CCR5: CC chemokine receptor 5
TLC: thin‐layer chromatography

## 1 Introduction

Lipid‐based nanoparticles (LNPs) have evolved from first‐generation liposomes into a versatile delivery platform that now supports mRNA vaccines, RNA‐interference medicines, long‐acting small‐molecule depots, and intracellular imaging agents [1, 2, 3]. The LNP family extends beyond liposomes to include nanostructured liquid and solid particles. Solid lipid nanoparticles have matured from a conceptual replacement for oil‐in‐water emulsions into a versatile nanoplatform that combines the regulatory familiarity of lipids with the colloidal control required for modern drug delivery [4]. Unlocking their full potential, however, demands a deeper, quantitative understanding of how composition, shape, size distribution, and internal architecture govern nano‐bio interactions and therapeutic performance [5, 6]. Typical solid LNPs are 50–300 nm dispersions produced by high‐pressure homogenization or melt emulsification and stabilized by non‐ionic or polymeric surfactants [7]. Their fully or partially crystalline cores confer long‐term physical stability, protect chemically labile drugs, and enable manufacturing without the use of organic solvents [8]. Yet the same crystallinity reduces lattice defects, making high drug load and controlled release difficult, but the loss of payload during cooling or storage remains the dominant limitation [9].

A widely adopted strategy to overcome this limitation is to blend a high‐melting solid lipid with a low‐melting liquid lipid or functional oil. Carnauba wax, a complex mixture of long‐chain esters that melts at 81–86°C and is already used in food and topical products, has proven particularly attractive [10]. When combined 1:1 with liquid esters or triglycerides, the melting enthalpy of the matrix drops from approximately 160 to 70 J g^−^^1^, indicating a less‐ordered lattice that can accommodate more guest molecules without compromising colloidal stability [10]. Such binary matrices routinely reach entrapment efficiency > 90% for hydrophobic actives and remain physically stable for almost 30 days, even under accelerated conditions [11].

Particle morphology adds another, often overlooked level of control. In practice, wax‐oil LNPs have been reported as anisotropic‐shaped particles by small‐angle X‐ray scattering (SAXS) [12], features that correlate with rapid cellular internalization and depot‐like behaviour in skin. Shape engineering, therefore, offers a complementary handle to composition and size distribution.

Against this backdrop, the particles chosen for this study are LNPs with a purpose‐built binary matrix composed of carnauba wax (solid) and red palm oil (liquid) stabilized by a mixed surfactant shell of d‐α‐tocopheryl PEG 1000 succinate (TPGS) and polysorbate 40 with the commercial name Cellinject [13]. The phase‐inversion protocol yields nanoparticles with hydrodynamic diameters of 35–55 nm, sizes already within the parenteral window for subcutaneous or intradermal injection. The carnauba wax furnishes mechanical integrity, while the unsaturated triglycerides of red palm oil introduce lattice imperfections analogous, maximizing free volume for guest molecules.

These LNPs show two distinct properties. First, the particles display *zero* detectable release of encapsulated fluorophore in aqueous media; payload liberation is initiated exclusively after cellular uptake and enzymatic erosion of the matrix. Second, short‐term cytotoxicity assays in HaCaT keratinocytes show good viability for 0.5% dispersions, underlining the biocompatibility of the lipid‐surfactant combination. Confocal imaging in HeLa cells further confirms rapid internalization and nucleolar accumulation of the released dye within 60 min, with fluorescent signal persisting for > 13 h [13].

The small size and low PDI achieved for these LNPs align with parameter optimization studies on carnauba‐based systems, in which processing and formulation parameters strongly control particle size and dispersity [10]. Here, the phase‐inversion approach, together with the additional fluidization provided by red‐palm oil, drives the mean diameter into the sub‐50 nm regime without broadening the distribution, an essential prerequisite for reproducible injectability through fine‐gauge needles. In classic solid LNPs, drug expulsion accompanies lipid recrystallization [9]. The disrupted lattice of the LNPs investigated in this study resembles nanostructured lipid carriers and supports high association efficiencies (>90%) even after four weeks at 4°C, as shown previously for rosmarinic‐acid‐loaded wax particles [11]. The absence of premature release in phosphate‐buffered saline (PBS) or culture medium underscores the tight molecular fit between guest and matrix. In addition, carnauba wax contributes not only a high melting point but also oxidative robustness, an advantage for unsaturated payloads [10].

Subcutaneous injections demand carriers that resist aggregation at high lipid concentrations, traverse the extracellular matrix without burst release, and degrade into biocompatible components. A narrow size distribution and sizes below 50 nm minimize syringe force and needle clogging. At the same time, the wax core slows erosion to create a local depot analogous to long‐acting polymer implants but without acidic degradation products. In skin models, carnauba‐based carriers form an occlusive lipid film that limits transepidermal water loss and enhances retention in the upper dermis [10], suggesting that injectable depots could similarly localize the drug near the injection site.

We harness orthogonal analytics—advanced asymmetrical flow field‐flow fractionation with multiple detectors (AF4‐MD), dynamic (DLS) and static light scattering (MALS), and fluorescence spectrometer as well as batch SAXS and cryogenic transmission electron microscopy (Cryo‐TEM)—to dissect how each compositional element (solid‐liquid lipid ratio, surfactant identity, payload level) shapes particle size, polydispersity, morphology and stability (see Tables 1 and 2).

**TABLE 1:** The composition of the LNPs containing a drug.

Compounds	Amount in w/w parts
Carnauba wax	1.00 to 5.00
Red palm oil concentrate (30% tocotrienols)	0.10 to 0.50
d‐a‐Tocopheryl polyethylene glycol 1000 succinate (TPGS)	0.70 to 3.50
Polysorbate 40	0.50 to 2.50
Drug^a^	q.s.
NaCl	0.9
Water	up to 100.0

^a^ Drug (quinine or dihydroartemisinin); q.s. quantum sufficient.

**TABLE 2:** Different batches (B) of the LNP formulation are summarized.

Sample	Description
LNP_5_ LNP_5_‐B2 LNP_5_‐B3 LNP_5_‐B4	Unloaded lipid nanoparticles dispersed in 0.9% NaCl solution, 5.5% lipid fraction, with carnauba wax and red palm oil as lipid core, with both TPGS and Polysorbate 40 as surfactants.
LNP_5_‐WS LNP_5_‐B2‐WS LNP_5_‐B3‐WS LNP_5_‐B4‐WS	Unloaded lipid nanoparticles dispersed in 0.9% NaCl solution, 5.5% lipid fraction, with carnauba wax and red palm oil as lipid core, with both TPGS and Polysorbate 40 as surfactants. Washed from residual surfactant.
LNP_5_‐Q_10_ LNP_5_‐B2‐Q_10_	Quinine (Q)‐loaded lipid nanoparticles dispersed in 0.9% NaCl solution, 5.5% lipid fraction, with carnauba wax and red palm oil as lipid core, with both TPGS and Polysorbate 40 as surfactants. 10% (w/w) quinine in lipid.
LNP_5_‐B4‐Q_20_	Quinine (Q)‐loaded lipid nanoparticles dispersed in 0.9% NaCl solution, 5.5% lipid fraction, with carnauba wax and red palm oil as lipid core, with both TPGS and Polysorbate 40 as surfactants. 20% (w/w) quinine in lipid.
LNP_5_‐DHA_3_ LNP_5_‐B4‐DHA_3_	Dihydroartemisinin (DHA)‐loaded lipid nanoparticles dispersed in 0.9% NaCl solution, 5.5% lipid fraction, with carnauba wax and red palm oil as lipid core, with both TPGS and Polysorbate 40 as surfactants. 3% (w/w) DHA in lipid.
LNP_5_‐B2‐DHA_5_	Dihydroartemisinin (DHA)‐loaded lipid nanoparticles dispersed in 0.9% NaCl solution, 5.5% lipid fraction, with carnauba wax and red palm oil as lipid core, with both TPGS and Polysorbate 40 as surfactants. 5% (w/w) DHA in lipid
LNP_1_	Unloaded lipid nanoparticles dispersed in 0.9% NaCl solution, 1.1% lipid fraction, with carnauba wax and red palm oil as lipid core, with both TPGS and Polysorbate 40 as surfactants.
LNP_1_‐B2 LNP_1_‐B3	Unloaded lipid nanoparticles dispersed in 0.9% NaCl solution, 1.2% lipid fraction, with carnauba wax and red palm oil as lipid core, with both TPGS and Polysorbate 40 as surfactants.
LNP_1_‐W	Unloaded lipid nanoparticles dispersed in ultrapure water, 1.1% lipid fraction, with carnauba wax and red palm oil as lipid core, with both TPGS and Polysorbate 40 as surfactants.
LNP_1_‐Q_10_	Quinine (Q)‐loaded lipid nanoparticles dispersed in 0.9% NaCl solution, 1.1% lipid fraction, with carnauba wax and red palm oil as lipid core, with both TPGS and Polysorbate 40 as surfactants. 10% (w/w) quinine in lipid
LNP_1_‐B3‐Q_8_	Quinine (Q)‐loaded lipid nanoparticles dispersed in 0.9% NaCl solution, 1.2% lipid fraction, with carnauba wax and red palm oil as lipid core, with both TPGS and Polysorbate 40 as surfactants. 8.3% (w/w) quinine in lipid
LNP_1_‐TPGS	Unloaded lipid nanoparticles dispersed in ultrapure water, 1.1% lipid fraction, with carnauba wax and red palm oil as lipid core, with TPGS as surfactant.
LNP_1_‐CW‐TPGS	Lipid nanoparticles dispersed in ultrapure water, 1.1% lipid fraction, with carnauba wax as lipid core, with TPGS as surfactant.
LNP_1_‐T40	Unloaded lipid nanoparticles dispersed in ultrapure water, 1.1% lipid fraction, with carnauba wax and red palm oil as lipid core, with Polysorbate 40 as surfactant.
LNP_1_‐CW‐T40	Lipid nanoparticles dispersed in ultrapure water, 1.1% lipid fraction, with carnauba wax as lipid core, with Polysorbate 40 as surfactant
LNP_1_‐FL	Fluorescein‐loaded lipid nanoparticles dispersed in 0.9% NaCl solution, 1.1% lipid fraction, with carnauba wax and red palm oil as lipid core, with both TPGS and Polysorbate 40 as surfactants, 0.45% (w/w) fluorescein in lipid

We then correlate these physicochemical fingerprints with in vitro cytocompatibility and ex vivo skin penetration to delineate design rules for under‐skin therapeutics. The resulting insights will inform not only the formulation of the LNPs, but also the broader development of wax‐based LNPs for localized under‐skin delivery.

## 2 Results and Discussion

### 2.1 Impact of Composition on Size and Stability

**FIGURE 1:**
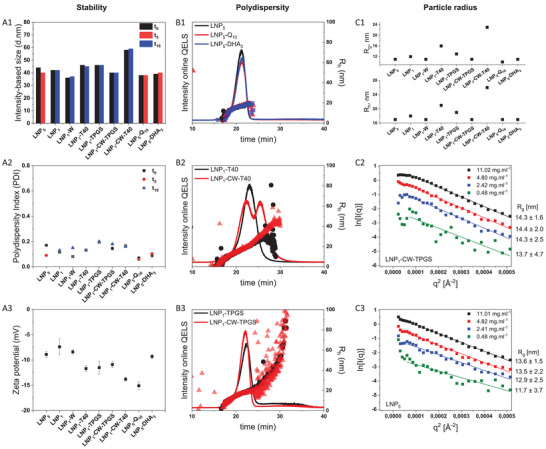
Impact of LNP composition on stability, dispersity and overall size. (A1) Dynamic light scattering (DLS) in batch determined intensity‐based particle size (diameter) of various LNP composition formulations as a function of time over 5 and 18 months confirms stability at 4°C. (A2) Polydispersity index (PDI) of various LNP composition formulations as a function of time, determined using DLS. (A3) Zeta (ζ) potential values for the various LNP formulations. (B1) AF4‐MD analysis of the size distribution of LNP without drug incorporation (LNP_5_) and with drug incorporation (LNP_5_‐Q_10_ and LNP_5_‐DHA_3_) are compared. (B2) AF4‐MD based comparison of two LNP formulations with the same polysorbate 40 surfactant, but different cores. LNP_1_‐T40 contains a mixture of carnauba wax and red palm oil, whereas LNP_1_‐CW‐T40 consist only of a carnauba wax core. (B3) AF4‐MD based comparison of two LNP formulations with the same TPGS surfactant, but different lipid cores. LNP_1_‐TPGS contains a mixture of carnauba wax and red palm oil, whereas LNP_1_‐CW‐TPGS consists only of a carnauba wax core. (C1) AF4‐MD‐based evaluation of the average R_h_ and R_g_ for different LNP composition formulations after separation. (C2) The Guinier plot from SAXS in batch evaluates the global R_g_ for four different concentrations of LNP_1_‐CW‐TPGS in water. (C3) The Guinier plot from SAXS in batch evaluates the global R_g_ for four different concentrations of LNP_5_ in water.

Engineering LNPs requires an understanding of the relationship between formulation and structural modification of size, shape and stability. To determine how lipid and surfactant composition controls nanoparticle size, polydispersity and long‐term colloidal stability, we combined an ensemble of complementary techniques. Batch dynamic light scattering (DLS) and small‐angle X‐ray scattering (SAXS) provide average hydrodynamic (R_h_) and gyration (R_g_) radii, while asymmetric flow field‐flow fractionation with multidetector (AF4‐MD) readout resolved the full‐size heterogeneity (Figure 1).

Batch DLS measurements of the average R_h_ vs storage time of up to 18 months (4°C) are plotted in Figure 1A1. Independent of the formulation, most particles remain within ±10% of their initial size, confirming good stability. The accompanying polydispersity indices (Figure 1A2) stay below 0.20 for all samples, indicating narrow size distributions and no time‐dependent broadening. LNP_5_’s modest fall in PDI suggests altered uniformity, whereas the small rise for LNP_1_‐W underlines the role of the dispersion medium in particle stability. For more information, see Tables S1 and S2. For selected formulations, independent batches prepared with the same phase‐inversion protocol showed good reproducibility (CV%), with batch‐to‐batch variations in mean size generally < 5% across all formulations (Table S2).

Zeta (ζ) potential measurements confirm sufficient electrostatic repulsion with −8 mV to −15 mV (Figure 1A3; Table S3). These modest negative values are sufficient to hinder aggregation, a conclusion supported by the absence of self‐association even at 11 mg mL^−^^1^ and in ultrapure water, corroborated also by SAXS with the absence of upturns at low q‐values (Figure 1C2,C3; Table S4). Drug loading affected the ζ‐potential: unloaded LNP_5_ measured −8.9 ± 0.6 mV, whereas quinine‐loaded LNP_5_‐Q_10_ registered −15.1 ± 0.8 mV, implying that quinine contributes additional negative charge and thereby enhances colloidal stability.

Because batch DLS can mask minor populations, we next use AF4‐MD to dissect size distributions more precisely (Figure 1B1–B3, see also Table S5 and Figures S1–S12). AF4 separation is essential for a complete view of particle heterogeneity, which here is derived from combined online DLS, multi‐angle light scattering (MALS) and UV–vis signals. Online DLS gave R_h_ values between 17 and 26 nm, slightly smaller than batch DLS because larger, more highly scattering populations dominate the batch measurement. The optimized AF4 method showed good robustness and reproducibility, with an average recovery of >80% (>95% for LNP_5_, see Table S6) and a relative standard deviation (RSD) below 5% for retention time, R_h_, and peak area across the various formulations. Furthermore, batch‐to‐batch consistency was also within range, with R_h_ RSD value remaining below 3% (Tables S6–S8). These values are well within the recommendations for nanoparticle characterization and translational readiness [14, 15].

Drug‐free LNP_5_ as well as its quinine‐ (LNP_5_‐Q_10_) and dihydroartemisinin‐loaded (LNP_5_‐DHA_3_) counterparts show a single, well‐defined elution peak of comparable width, yielding monomodal R_h_ distributions (<17 nm ± 0.2 nm) (Figure 1B1), confirming that drug incorporation does not alter particle size. In contrast, lipid composition influences the observed size distribution: removing red‐palm oil (LNP_1_‐CW‐T40) leads to a bimodal profile, whereas the mixed‐lipid analogue (LNP_1_‐T40, carnauba wax and red‐palm oil) exhibits a unimodal distribution within a single broad peak (Figure 1B2). R_h_ values above 20 nm across both peaks of LNP_1_‐CW‐T40 indicateincreased heterogeneity relative to LNP_1_‐T40, a formulation in which red palm oil is present.

Surfactant choice is also associated with changes in size distribution: TPGS‐only systems show broad distribution irrespective of the lipid composition investigated, as demonstrated on LNP_1_‐TPGS (mixed lipids) and LNP_1_‐CW‐TPGS (carnauba wax only). These formulations showed single peaks with pronounced tailing toward > 80 nm and broad R_h_ ranging from 14 nm to over 100 nm across the elution peak, in contrast to the dual‐surfactant combination (Polysorbate 40 and TPGS, as in LNP_5_), which shows a well‐defined size and distribution.

Overall, the AF4‐MD results indicate that omission of red‐palm oil (LNP1‐CW‐T40, LNP1‐CW‐TPGS) is associated with broader or bimodal elution profile, whereas the carnauba‐wax/red palm oil matrix formulations exhibit a dominant monomodal population, suggesting a potential stabilizing influence of the oil in narrowing the R_h_. Taken together, these comparisons indicate that both the mixed wax‐oil matrix and the mixed surfactant combination are associated with suppressing bimodality and limiting the population of larger particles, while a single surfactant appears sufficient to achieve basic colloidal stability. When lipid composition is fixed, TPGS‐containing particles (LNP_1_‐TPGS) elute as a single mode, yet with smaller R_h_ values than the Polysorbate 40 formulation (LNP_1_‐T40).

Absolute dimensions from AF4‐MALS (Figure 1C1) matched the average R_g_ extracted from SAXS. Guinier analysis across four concentrations gave R_g_ = 14 ± 2 nm for LNP_1_‐CW‐TPGS and 13 ± 2 nm for LNP_5_, confirming essentially the same dimensions. These results verify that both systems are aggregate‐free in both water and 0.9% NaCl (Figure 1C2,C3; Figure S21).

In summary, only formulations containing both lipids and both surfactants maintain a unimodal distribution that remains stable upon drug loading, whereas omitting any component results in a broader or even a split in the size distribution. Having established the compositional rules for producing stable, monodisperse particles, we next examined how these same variables sculpt particle shape and internal architecture.

### 2.2 Shape and Internal Composition

**FIGURE 2:**
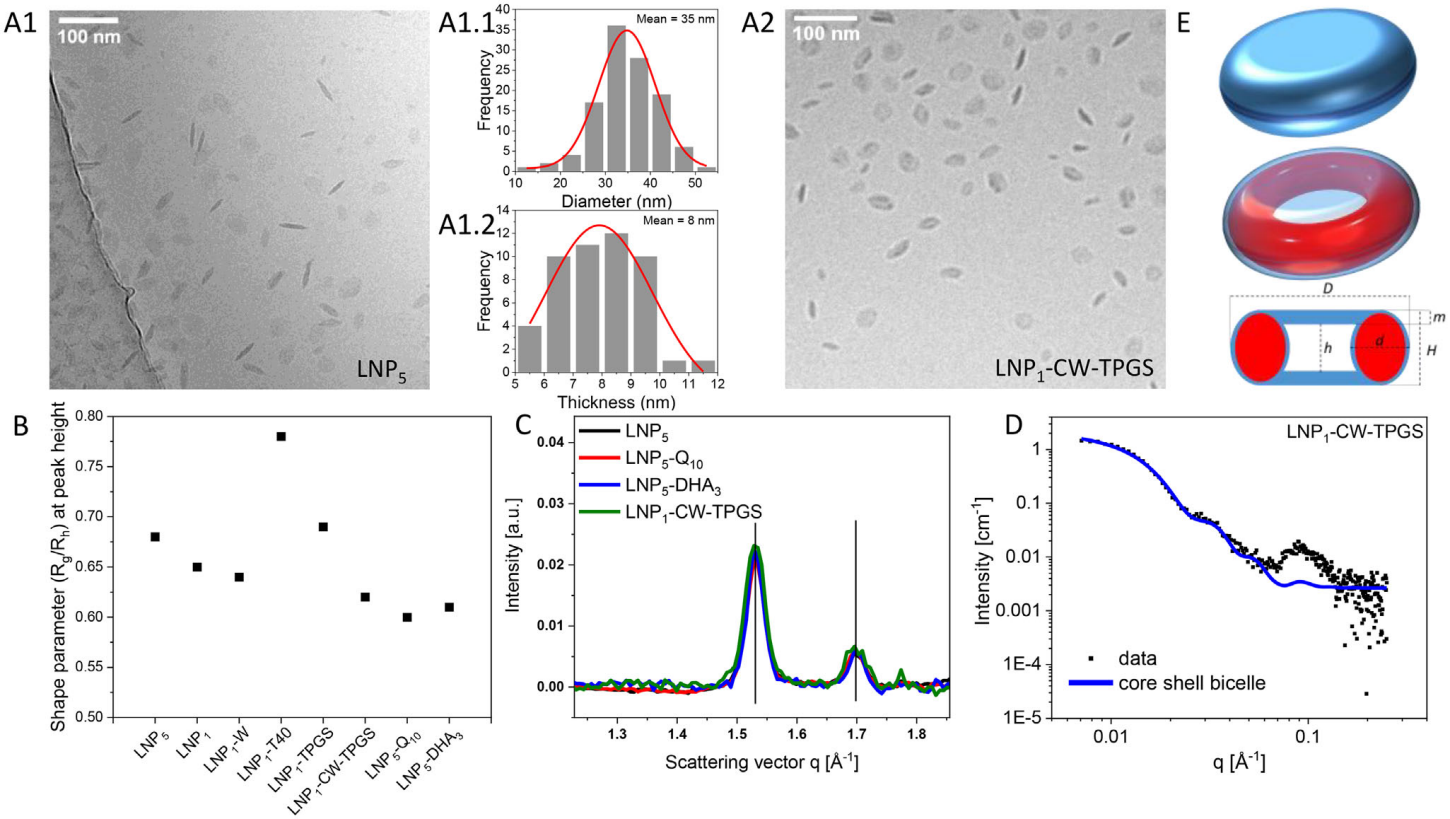
Shape and internal composition evaluation. (A1) Cryo‐TEM image of LNP_5_ (Unloaded LNP with carnauba wax and red palm oil as lipid core, with both TPGS and Polysorbate 40 as surfactants), (A1.1) the average particle size distribution, and (A1.2) the average particle thickness distribution. (A2) Magnified cryo‐TEM image of LNP_1_‐CW‐TPGS (Unloaded LNP with carnauba wax only lipid core and TPGS as surfactant) to provide a clearer view of the observed shape. (B) Shape parameter R_g_/R_h_ determined at the apex of the UV signal  for the various compositions of LNP formulation using AF4‐MD show values corresponding to spherical shapes. (C) WAXS plots of LNP_5_, LNP_5_‐Q_10_, LNP_5_‐DHA_3_ and LNP_1_‐CW‐TPGS. (D) SAXS data and modelling by a core‐shell bicelle for LNP_1_‐CW‐TPGS in water at a concentration of 11.02 mg mL^−1^ based on lipid fraction. The geometry parameters correspond to the values described in the sketch under (E) with D = 32.6 ± 0.4 nm; d = 8.9 ± 0.1 nm; h = 8.2 ± 0.2 nm; H = 14.6 ± 0.4 nm; m = 3.2 ± 0.1 nm. For the calculation, the SLD for water (core) and lipid (internal toroid) were applied. (E) Schematic representation of the particle shape corresponding to a disk‐toroid hybrid based on structural insights gained from the experimental data using cryo‐TEM and SAXS: the internal lipid toroid is stabilized by a monolayer of surfactant while the water core is stabilized by a double layer of surfactant at the interface between the core and aqueous particle environment.

To that end, we combined high‐resolution cryogenic transmission electron microscopy (cryo‐TEM) with AF4‐based shape analysis and multiscale X‐ray scattering (Figure 2).

Cryo‐TEM captures the LNPs in their fully hydrated state, eliminating artifacts from drying or staining. The micrographs for LNP_5_ (Figure 2A1) and LNP_1_‐CW‐TPGS (Figure 2A2) reveal two dominant morphologies: ellipsoidal with 30–40 nm in diameter (Figure 2A1.1), yet a fraction exhibits toroidal contrast, hinting at compositional variations within the core; and more elongated, high‐contrast objects (thickness ≈ 7–9 nm; Figure 2A1.2). More micrographs of the LNPs are available in Supplementary Information (see Figures S14–S19). In fact, these two morphologies belong to the same structure, as a 3D rendering unveils (see Figure S20) and confirms an overall disc‐like shape irrespective of composition. To probe topology, crystallinity, and shape in greater detail, AF4 and X‐ray scattering were employed.

To quantify this observation, we extracted the shape parameter (R_g_/R_h_) at the apex of each UV signal (Figure 2B; Table S9). Values of 0.60 – 0.78 fall below the hard‐sphere limit (0.78), and far below the ∼2.0 expected for flat discs [16, 17, 18, 19], indicating a complex, non‐spherical architecture whose nuances require higher‐resolution probes.

To shed light on the internal structure, wide‐ and small‐angle scattering (WAXS and SAXS) were applied. WAXS interrogated crystallinity: Two reflections at q_1_ = 1.53 Å^−^^1^ and q_2_ = 1.70 Å^−^^1^ correspond to carnauba‐wax spacings d_1_ = 0.41 nm and d_2_ = 0.37 nm (Figure 2C) [20]. Drug loading with quinine or artemisinin leaves both lattice distances and crystallinity unchanged, suggesting the drugs reside outside the crystalline domains, presumably near the particle surface.

SAXS resolved the internal topology: For LNP_1_‐CW‐TPGS (Figure 2D) and LNP_5_ (Figure S22) at ∼ 11 mg mL^−^^1^, lipid differences arise mainly between q = 0.025 and 0.05 Å^−^^1^. A core‐shell bicelle model, featuring distinct scattering length densities (SLDs) for core and shell, fits the data best (χ^2^ < 7.3), while spherical and ellipsoidal models fail. A sharp peak at q = 0.09 Å^−^^1^ remains unaccounted for by the bicelle model, but matches the signature of surfactant bilayer (∼7 nm) reported for Polysorbate‐80 systems [12].

Shell SLDs were fitted to 9 × 10^−^^6^ Å^−^^2^, whereas core SLDs are higher with 9.461 × 10^−^^6^ Å^−^^2^ (LNP_1_‐CW‐TPGS) to 9.225 × 10^−^^6^ Å^−^^2^ (LNP_5_). Considering that water has SLD = 9.47 × 10^−^^6^ Å^−^^2^, the increased core SLD implies a water‐rich interior—an almost empty core enclosed by a lipid shell and a surfactant bilayer. This agrees with previous SAXS reports on solid LNPs that found similar SLD parity between water and surfactant layers [12]. Modeled dimensions (Figure 2E) align with the shape found by cryo‐TEM, though SAXS yields an average aspect ratio of 2:1 versus 4:1 from cryo‐TEM. This difference stems from the fact that SAXS is a batch method delivering an average size over the complete sample populations, while cryo‐TEM gives a particle‐by‐particle view covering a small fraction of these populations.

The observed toroidal geometry enlarges the surface‐to‐volume ratio relative to an equivalent‐volume sphere, which is expected to favor drug diffusion and extensive membrane interaction [21]. Consistent with this interpretation, differential scanning calorimetry of wax‐oil matrices shows lowered enthalpy and onset temperatures, signatures of less‐ordered α/β′ crystals [10], while the asymmetric TPGS/Polysorbate‐40 shell promotes curvature. Together, these factors explain the torus‐like SAXS patterns seen in wax nanoparticles and may accelerate enzymatic access after injection. This provides a plausible structural basis for the rapid intracellular disassembly and depot‐like behavior observed in previous Cellinject [13], however geometry‐specific effects can only be confirmed through dedicated mechanistic experiments.

### 2.3 Drug Encapsulation—Quantification and Localization

**FIGURE 3:**
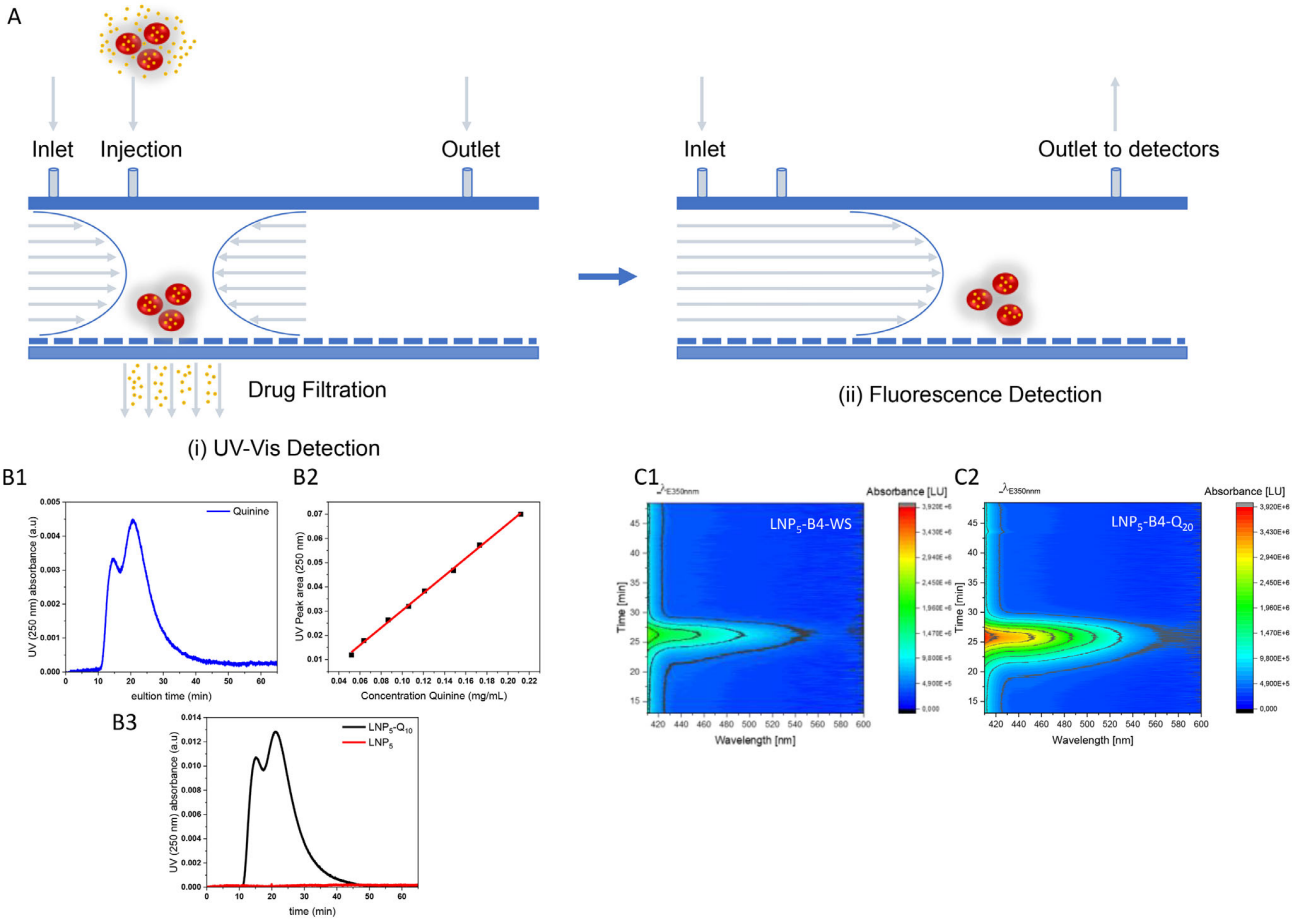
Drug encapsulation and quantification. (A) A schematic representation illustrating two alternative approaches for the indirect determination of the encapsulation efficiency of LNP, using AF4 coupled to either a UV–vis detector or a fluorescence detector. A collection of small drug molecules filtered through the membrane of the AF4 channel is quantified using a pre‐calibrated UV–vis detector. The large drug‐loaded particles separated along the channel are detected using the fluorescence detector. (B1) Elution profile showing the UV–vis signal of quinine, with the cross‐flow coupled to the UV–vis detector. Wavelength set at 250 nm. (B2) The calibration curve is generated by injecting several concentrations of quinine, enabling the quantification of quinine present in the cross‐flow waste. Wavelength set at 250 nm. (B3) Elution profile showing the UV–vis signal of LNP_5_ and LNP_5_‐Q_10_, respectively. The cross‐flow is coupled to the UV–vis detector, set to a wavelength of 250 nm. (C1) and (C2) Size‐based fluorescence detection of the LNPs indicates that quinine is located within the particles, as evidenced by the increased fluorescence emission intensity at the same elution volume as the particle peak. The particle concentration is kept constant; therefore, the higher fluorescence emission intensity and the broader emission spectrum (after excitation at 350 nm) observed for the loaded LNP_5_‐B4‐Q_20_ (C2) compared to the pure LNP_5_‐B4‐WS (C1) can be attributed to the presence of quinine.

With particle architecture defined, we turned to the next question: how much drug do the bicellar LNPs carry, and where does it reside? As a single‐run solution. AF4‐MD is uniquely suited to deconvolute conformation, size, molar mass, and composition distributions of heterogeneous nanoparticles across a broad size spectrum (Figure 3A) [16]. The channel's semi‐permeable membrane, here 10 kDa, acts as a molecular sieve and defines the lower size limit of AF4 separation: species smaller than the cut‐off permeate the membrane and are swept into the cross‐flow line instead of being separated in the channel. What might appear to be a drawback becomes an efficient way to quantify free drug passing through the membrane [22, 23]. We therefore quantified the non‐encapsulated quinine fraction from a calibrated UV signal collected at the cross‐flow outlet. At the same time, an in‐line fluorescence detector traced the drug associated with the LNP carrier.

A dual readout thus leads to dual information:

1. UV at the cross‐flow outlet: The cross‐flow waste is routed directly to a UV–vis detector (Figure S13 and Table S10). Any free drug that has diffused through the membrane appears as a UV peak. Calibration created using drug standards (Figures 3B1,B2) relates peak area to concentration. A wavelength of 250 nm was selected because unloaded LNP_5_ shows no absorbance at this wavelength (Figure 3B3), whereas the quinine‐loaded sample (LNP_5_‐Q_10_) yields a clear signal. Integrating that peak and subtracting it from the total drug added during LNP formation gives an encapsulation efficiency of ∼90%. The non‐encapsulated (free‐drug) yielded a peak area corresponding to concentrations well above the calculated LOQ (0.0152 mg mL^−1^), indicating that the quantification of the non‐encapsulated fraction is robust (LOD = 0.005 mg mL^−1^, Table S11). This calculation is in full agreement with the data from the recovery measurements and the determined sample loss during the focusing step (Table S6).
2. In‐line fluorescence spectroscopy: Quinine and unloaded LNPs have different fluorescence maxima, while the quinine‐loaded particles emit at wavelengths close to quinine (Figure S23). Batch measurements alone cannot reveal whether quinine is incorporated into the LNPs, but AF4‐coupled fluorescence spectroscopy resolves the ambiguity. Overlapping elution profiles, higher fluorescence intensity at equal concentrations, and the red‐shifted maximum prove that quinine is bound to the nanoparticles rather than present as a separate free species (Figure 3C1,C2).

Through this orthogonal UV and fluorescence analysis, we demonstrate that ≈90% of the added quinine is associated with the bicellar LNPs, while the membrane‐permeable fraction remains minimal—an essential prerequisite for predictable dosing in downstream biological studies.

The AF4‐MD workflow establishes a multi‐parameter characterization platform for disc‐toroid LNPs, including size distribution, morphology (R_g_ and R_g_/R_h_), minor aggregate populations, and drug encapsulations, with recovery and repeatability metrics. This approach allows differentiation between predominantly unimodal particle populations and formulations with increased heterogeneity. While not a fully validated assay for monitoring critical quality attributes (CQA), the workflow demonstrates the feasibility of integrated CQA assessment and provides a basis for further methodological development and more detailed batch‐to‐batch consistency for later translational development of these LNP.

Taken together, the data so far paint a complete physicochemical picture of our solid‐liquid nanoparticles. We have shown that a dual‐lipid/dual‐surfactant formulation maintains a hydrodynamic radius at ≈17 nm for at least 18 months, which exhibits a bicelle type of architecture with an enlarged surface‐to‐volume ratio, and that ≈90% of the model drug is associated with that scaffold. With the particles’ composition, shape and loading capacity now firmly established, the obvious next question is whether these design advantages translate into biological benefit. In the following, we therefore examine, first, how morphology and surface chemistry dictate cellular uptake and compatibility, and second, how the loaded LNPs behave in tissue‐level models that mimic subcutaneous or intradermal administration—thereby closing the loop from formulation, through structure, to function.

### 2.4 Bioactivity in Human Cells

**FIGURE 4:**
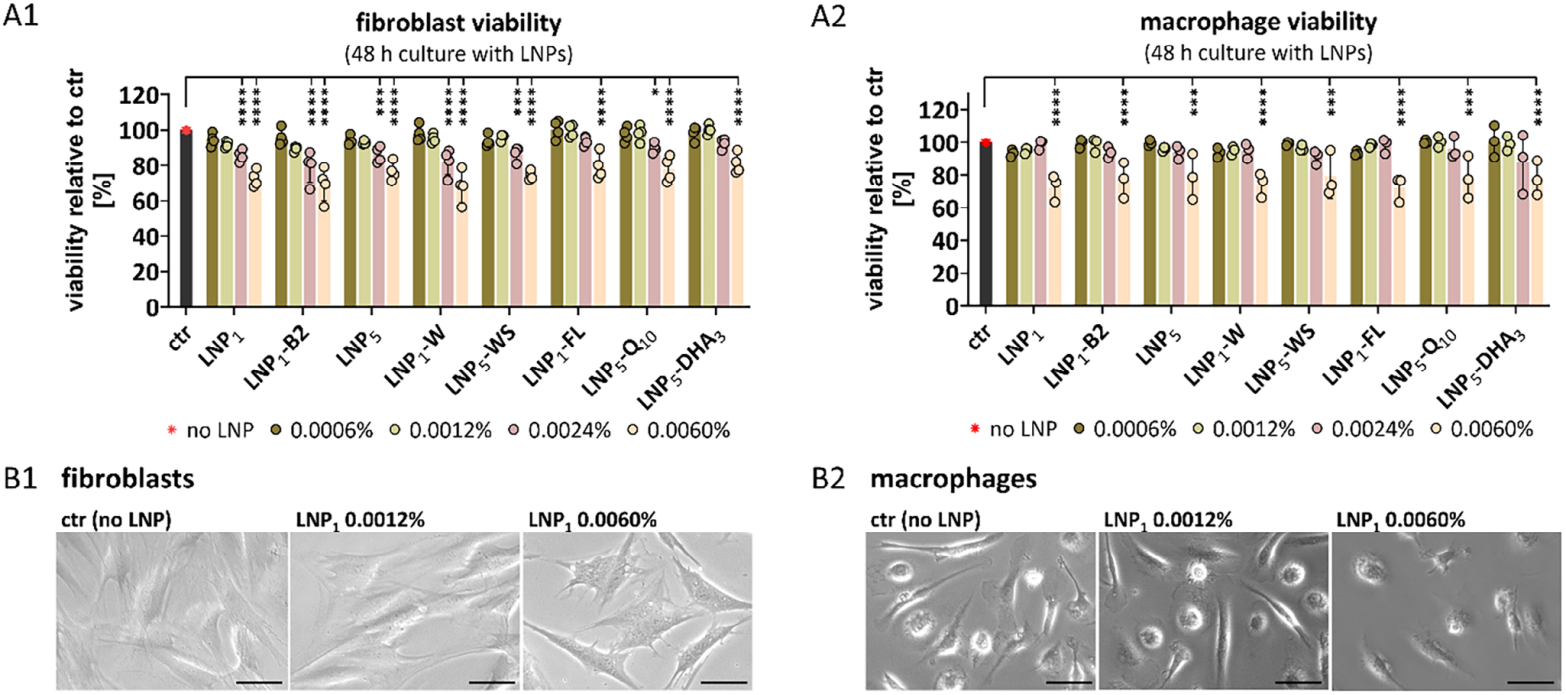
Cell viability after culture with LNP. (A, B) Monocyte‐derived human macrophages and human dermal fibroblasts were cultured for 48 h with different LNPs at different concentrations. LNP concentrations are indicated as % lipid fraction. LNP_1_, LNP_1_‐B2, and LNP_5_ comprise different batches of LNP. LNP_1_‐W are in pure water. LNP_5_‐WS, the residual surfactants were removed. LNP_1_‐FL, LNP_5_‐Q_10_, and LNP_5_‐DHA_3_ comprise LNP batches loaded with fluorescein (FL) or with the drugs quinine (Q) or dihydroartemisinin (DHA). (A1) Viability of 4 fibroblasts was determined with the XTT assay. n = 4 different fibroblast donors. (A2) Viability of macrophages was determined with the XTT assay. n = 3 different macrophage donors. (B1) and (B2) Microscopic evaluation of macrophages and fibroblasts. Scale = 50 µm. (A1/A2) Two‐way ANOVA with Tukey‘s multiple comparisons test. Significant differences compared to ctr (no LNP) are indicated. ^*^ *p* < 0.05, ^***^ *p* < 0.001, ^****^ *p* < 0.0001.

Intradermal or subcutaneous injection of LNPs is emerging as a promising approach for targeted local drug delivery, owing to their ability to provide sustained drug release over time. The biocompatibility of LNPs following skin injection appears to depend on the specific lipids and surfactants used in their formulation [13]. To investigate this further, we assessed the cytotoxicity of a range of LNP variants on primary human fibroblasts and macrophages as examples of skin‐resident tissue and immune cells that play key roles in skin homeostasis and repair. Fibroblasts produce the extracellular matrix (ECM) essential for skin structure, while macrophages function as immune sentinels and contribute to tissue regeneration. Their coordinated interaction is vital for maintaining skin integrity and responding to injury [24, 25]. Co‐culture of LNPs with fibroblasts and macrophages for 48 h demonstrated very good biocompatibility up to a lipid concentration of 0.0024% for fibroblasts (Figure 4A1) and up to 0.006% for macrophages (Figure 4A2). This high level of biocompatibility was maintained even after 7 days of co‐culture (Figure S24A,C). At higher lipid concentrations, occasional morphological signs of cellular stress and apoptosis were observed (Figure 4B1 and B2; Figure S24B,D), which corresponded to a slight but statistically significant reduction in cell viability. In contrast, different LNP dispersants, the presence of surfactants and drug loading of LNP had no measurable impact on cell viability (Figure 4A1,A2; Figure S24A,C).

**FIGURE 5:**
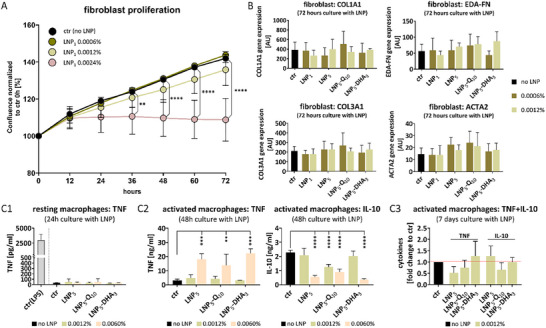
Effect of LNP on cell functions. (A) LNP_1_ at different concentrations were added to human dermal fibroblasts. Proliferation of fibroblasts was monitored via IncuCyte live cell imaging. *n* = 4. Two‐way ANOVA with Dunnett‘s multiple comparisons test. Significant differences compared to ctr are indicated. ^**^ *p* < 0.01, ^****^ *p* < 0.0001. (B) LNPs at different concentrations were added to human dermal fibroblasts, which were then stimulated with TGF‐β for 72 h. Expression of matrix genes COL1A1, COL3A1, EDA‐FN and myo‐fibroblast activation gene ACTA2 were quantified. TGF‐β‐stimulated fibroblasts without LNP served as control. *n* = 3. (C) Monocyte‐derived human macrophages were cultured with LNPs at different concentrations. Culture without LNP served as a control. Macrophage activation was induced by stimulation with LPS 24 h before the end of culture. (C1) TNF release of resting macrophages after 24 h culture with LNPs. LPS‐stimulated macrophages serve as a stimulation control (ctr‐LPS). *n* = 4. (C2) Release of TNF and IL‐10 from activated macrophages after 48 h culture with LNPs. *n* = 4. (C3) Release of TNF and IL‐10 from activated macrophages after 7 days culture with LNPs. Released cytokines are presented as fold change to control. *n* = 4. (B/C1/C2/C3) Two‐way ANOVA with Tukey‘s multiple comparisons test. Significant differences compared to ctr are indicated. ^*^ *p* < 0.05, ^**^ *p* < 0.01, ^***^ *p* < 0.001, ^****^ *p* < 0.0001. (A–E) LNP concentrations are indicated as % lipid fraction. LNP_1_, LNP_5_, LNP_5_‐Q_10_, and LNP_5_‐DHA_3_ comprise different batches of LNP as outlined in Figure 4.

To further assess the cytocompatibility of LNPs, we investigated their impact on key fibroblast functions in tissue homeostasis, such as cell growth, and in tissue repair, such as extracellular matrix (ECM) production and myofibroblast activation [26], here simulated through stimulation with TGF‐β. Consistent with viability assays, no effects on fibroblast proliferation were observed up to a lipid concentration of 0.0012%, whereas higher concentrations impaired proliferation (Figure 5A; Figure S25A). Neither the use of different LNP batches nor drug loading influenced fibroblast proliferation (Figure S25B). Accordingly, LNPs at a lipid concentration of 0.0012% had no impact on fibroblast function, as evidenced by unchanged expression levels of key ECM components COL1A1, COL3A1, EDA‐FN (fibronectin) and the myofibroblast marker ACTA2 (α‐smooth muscle actin) (Figure 5B).

To evaluate the potential immunogenic effects that LNPs would induce after application in the skin [27], we analyzed cytokine responses in resting macrophages after LNP exposure. No induction of TNF‐α—a representative pro‐inflammatory cytokine macrophages produce in response to their activation [28], for example, with LPS—was detected after 48 h of co‐culture (Figure 5C1), nor after 7 days (data not shown). We further examined whether LNPs modulate inflammatory responses in pre‐activated macrophages and exemplarily analyzed the release of TNF as an inflammatory macrophage signal, and the release of IL‐10 as a macrophage‐derived signal for inflammatory resolution [28, 29]. Consistent with viability data, LNP concentrations of 0.0012% had no effect on cytokine release after 48 h or 7 days of co‐culture (Figure 5C2,C3). However, for LNPs with 0.006% lipid concentrations, a marked increase in TNF‐α production, along with a decrease in the anti‐inflammatory cytokine IL‐10, was observed (Figure 5C2), suggesting a concentration‐dependent pro‐inflammatory shift.

### 2.5 Biocompatibility in the Skin

Next, we assessed the behavior of lipid nanoparticles (LNPs) under more native‐like conditions by employing a human ex vivo skin culture model (Figure S26A) to investigate LNP biocompatibility in a biologically and physiologically relevant context [30]. The skin maintained structural integrity over a 14‐day culture period, with only minor epidermal detachment observed by day 14 (Figure S26B). Notably, the dermis and hypodermis remained well‐preserved, showing minimal and consistent levels of apoptosis throughout the culture period (Figure S26C). However, given the generally reduced gene expression observed in cultured skin compared with fresh skin, we further assessed the functional responsiveness of the skin model. Inflammatory challenge of the skin led to an upregulation of inflammatory cytokines, while extracellular matrix (ECM) deposition was reduced over the course of culture demonstrating that the skin model elicits tissue responses consistent with those observed in vivo (Figure S26D). Conversely, pro‐fibrotic stimulation induced increased ECM protein expression and deposition in the skin model (Figure S26E–G). In summary, our ex vivo skin culture model preserves tissue integrity and functional responsiveness for up to 14 days, consistent with findings from previous studies [31, 32], and provides a suitable platform for investigating potential adverse effects of LNPs within this timeframe.

**FIGURE 6:**
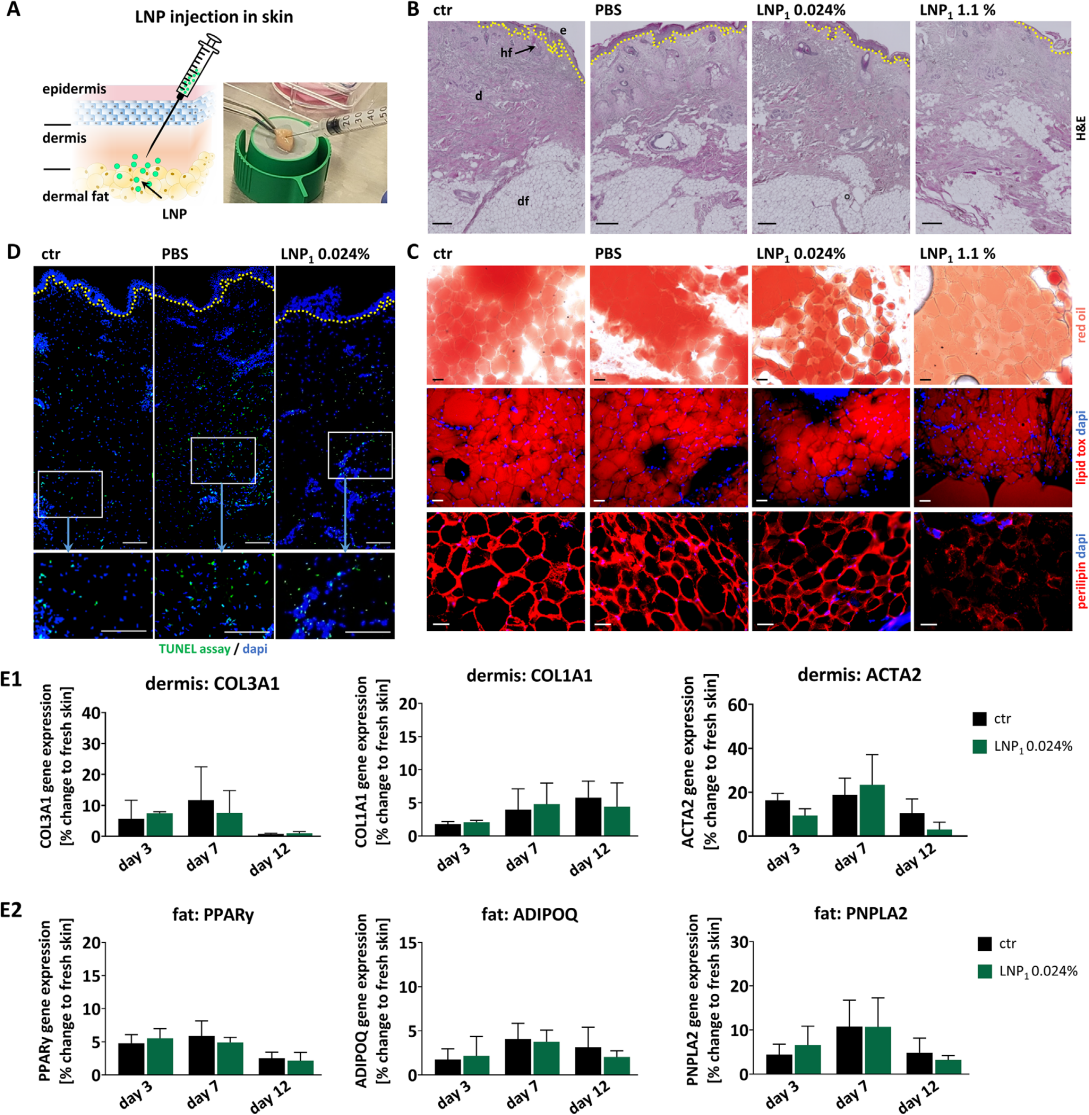
Biocompatibility of LNP in human skin. (A) Illustration of LNP injection into human skin. (B–E) 100 µL of LNP_1_ at 1.1% lipid fraction (LNP_1_ 1.1%) or of LNP_1_ diluted in PBS at 0.024% lipid fraction (LNP_1_ 0.024%) were injected in the deeper dermis/dermal fat area of human skin. Untreated human skin served as a control (ctr). Injection of 100 µL PBS served as a treatment control (PBS). Skin samples were cultured ex vivo as shown in Figure S26A. (B) Hematoxylin and Eosin (H&E) staining of histological skin sections. Skin culture ex vivo for four days. scale = 300 µm (C) Evaluation of lipolysis in the dermal fat by staining with red oil, lipid tox and perilipin in histological sections. Skin culture ex vivo for four days. scale = 50 µm. (D) Evaluation of skin viability via TUNEL staining of histological sections. Skin culture ex vivo for two days. scale = 200 µm. (E1) and (E2) Gene expression analysis of dermis and dermal fat isolated from different skin samples after culture ex vivo for three days, 7 days and 12 days. Data are presented as percent change compared to fresh skin. Fresh skin = 100% not shown. Statistical analysis was performed using two‐way ANOVA followed by Tukey's multiple comparisons test. No statistically significant differences were observed between the groups. *n* = 4.

LNPs at various lipid concentrations were injected at the dermis‐hypodermis interface, as illustrated in Figure 6A. Histological analysis of skin samples four days post‐injection revealed well‐maintained skin architecture. No differences were observed between untreated skin, PBS‐injected controls, and skin injected with LNPs at 0.024% lipid concentration (Figure 6B). However, skin injected with LNPs at 1.1% lipid concentration showed mild disruption in adipocyte integrity, suggesting lipolysis. Lipolysis is a key metabolic process in which lipids stored in fat cells are broken down into glycerol and fatty acids, typically in response to fat activation and stress [33, 34, 35].

The occurrence of lipolysis in skin injected with LNPs at 1.1% lipid concentration was supported by lipid staining (Red Oil and LipidTOX) and the observed loss of perilipin, a key lipid droplet‐associated protein [36] (Figure 6C). In contrast, adipocyte morphology remained intact in all other conditions. TUNEL assay confirmed good viability of skin injected with LNPs at 0.024%, with apoptosis levels comparable to PBS‐treated controls (Figure 6D). To assess potential functional changes in skin cells following LNP injection, dermal and fat layers were separated post‐culture for gene expression analysis. Although cultured skin showed an overall decrease in gene expression compared to fresh skin, no significant differences were observed between control and LNP‐injected skin (0.024%) in the expression of extracellular matrix‐related genes (COL1A1, COL3A1, ACTA2) in the dermis (Figure 6D). Similarly, genes related to adipogenesis, adipocyte activation and lipolysis (PPARγ, ADIPOQ, PNPLA2, respectively) [37, 38, 39] were not differentially expressed between control and LNP‐treated fat tissue (Figure 6D).

In summary, the evaluation of LNPs in the human ex vivo skin model demonstrated excellent biocompatibility at lipid concentrations up to 0.024%, with no structural or functional impairments observed at this dose. This dose is 40‐ to 160‐fold below the maximal lipid levels seen in approved LNP drugs. Thus, 0.024% lipid is high enough to carry a useful drug payload (e.g. a 5% w/w loading would deliver 12 µg drug in the 100 µL bolus), matching the micro‐ to low‐milligram doses often needed for depot‐type subcutaneous therapies [40]. And in addition, it remains well inside the safety envelope defined by marketed LNP products and by our own in‐vitro safety margins.

**FIGURE 7:**
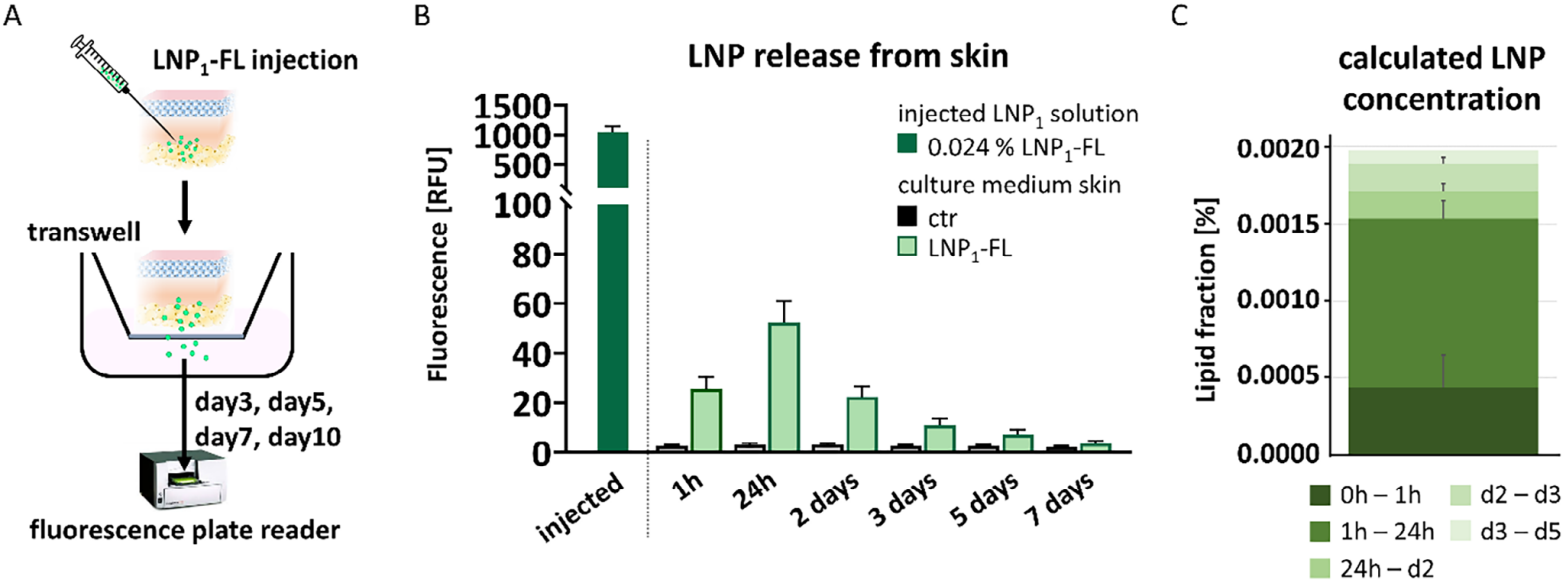
Penetration of LNP through the dermal and fat layer of human skin. (A) Illustration of experimental set‐up. (B/C) 100 µL of LNP_1_ loaded with fluorescein (LNP_1_‐FL) diluted at 0.024% lipid fraction was injected in the deeper dermis/dermal fat area of human skin. Untreated human skin served as control (ctr). Skin samples were placed in a transwell (0.4 µm pore size) in a culture plate and cultured ex vivo. After the indicated time points, the culture medium was replaced with fresh medium and subjected to fluorescence analysis. (B) Quantification of green fluorescence of the collected culture medium and the initially injected LNP_1_‐FL solution. *n* = 4. Statistical analysis was performed using two‐way ANOVA Šídák's multiple comparisons test. ^**^ *p* < 0.01, ^****^ *p* < 0.0001. (C) Calculated lipid fraction that accumulated in the medium during the culture periods of the skin injected with LNP_1_‐FL. *n* = 4.

Solid lipid nanoparticles are a major subclass of lipid‐based nanocarriers, particularly well‐suited for targeted drug delivery [41]. Effective drug delivery through the skin requires efficient penetration across its layers, which has been suggested to depend on specific physicochemical properties of the LNPs, such as solvent polarity and particle size [42]. To assess whether LNPs can achieve skin penetration, we injected fluorescein‐loaded LNPs at a lipid concentration of 0.024% into our human ex vivo skin model. The injected skin was placed in a transwell system with a membrane pore size that permits the passage of LNPs but not cells (Figure 7A). Supernatants were collected at defined time points following injection, and fluorescence intensity was measured using a plate reader. At each time point, the culture medium was fully replaced to ensure accurate quantification of continuous release. We observed already one hour after injection a fluorescence signal in the supernatant, which peaked at 24 h, followed by a gradual decline over subsequent time points (Figure 7B). It is noteworthy that the measured total fluorescence corresponds to a maximum lipid concentration of approximately 0.002% (Figure 7C). These findings indicate that a small but detectable fraction of LNPs was able to actively pass through the dermal and hypodermal layers.

In summary, our data suggest that LNPs can penetrate through the deeper layers of the skin in this model, supporting their potential for transdermal drug delivery. Our findings are supported by a previous study showing that lipid nanoparticles smaller than 100 nm fall within the optimal size range for deep skin penetration [43].

## 3 Conclusions

This study delineates a clear design space for solid‐liquid LNP depots and demonstrates that rational matrix and surfactant engineering yield carnauba‐wax/red‐palm‐oil lipid nanoparticles (LNPs) that combine long‐term colloidal stability with high drug‐loading capacity and excellent tissue compatibility.

Dispersions prepared by phase inversion remain monodisperse for at least 18 months at 4°C, retaining a hydrodynamic diameter below 50 nm and a polydispersity index ≤0.20. Formulations containing both the mixed TPGS/polysorbate‐40 corona and red palm oil exhibit a narrower size distribution, whereas omitting the red palm oil or using single‐surfactant systems can result in a broader size distribution and an increase in the relative presence of larger populations, even though a monomodal peak is observed under the analysis conditions. Systematic variation of surfactant ratios and total surfactant content will be important future work for translational development to fine‐tune interfacial curvature and biological interactions in a controlled manner. Quinine and dihydroartemisinin can be encapsulated at ≈ 90% efficiency without altering particle size or wax lattice order. Zeta potentials of −8 to −15 mV prevent aggregation even at lipid concentrations exceeding 10 mg mL^−^^1^, enabling highly concentrated injectable depots.

A distinctive disc‐toroid population that provides an enlarged surface‐to‐volume ratio has been confirmed, complemented by Cryo‐TEM and SAXS. The hybrid shape is not just a morphological curiosity: variation in surface area dictates how much membrane each particle can, in principle, contact and therefore influence cellular uptake. Particles with disc‐toroid geometry are expected to interact with cell membranes differently from their spherical counterparts, potentially modulating internalization kinetics. A systematic investigation of the impact on skin cell functions, specifically fibroblasts and macrophages, is therefore a logical next step to establish correlations between particle shape and biological fate.

Cytotoxicity thresholds lie well above therapeutically relevant lipid doses (≤0.006% for macrophages and ≤0.0024% for fibroblasts), and ex vivo human skin retains normal architecture and gene expression after injection of 0.024% lipid. Only trace amounts (≤0.002%) traverse the dermis within 24 h, confirming depot‐like behavior.

Collectively, these insights converge on a formulation window in which disc‐toroid LNPs combine long‐term physical stability, high drug payload and excellent tissue compatibility. They therefore constitute a versatile platform for minimally invasive delivery of small‐molecule or biopharmaceutical therapeutics. Future work should couple this structural blueprint with in vivo pharmacokinetics and ligand‐directed surfactant coronas to fully exploit their therapeutic potential.

## 4 Materials and Methods

### 4.1 Materials

Phosphate buffer saline (PBS) tablets (Sigma‐Aldrich); sodium azide (NaN_3_) (Sigma‐Aldrich), sodium chloride (NaCl) (Merck and Fisher Chemical), MilliQ ultrapure water, carnauba wax no.1 yellow (Acros organics), palmtocos 50 (red palm oil concentrate) (Gustav Parmentier GmbH), d‐a‐Tocopheryl polyethylene glycol 1000 succinate (TPGS) (Antares Health Products) and polysorbate 40 (Sigma‐Aldrich). Quinine (Sigma‐Aldrich) and dihydroartemisinin (Apidogen) were used as the payload. All materials were used as received unless otherwise stated.

### 4.2 Preparation of Unloaded and Loaded Lipid Nanoparticles

For the preparation of dispersion of the lipid nanoparticles (LNP), carnauba wax, red palm oil concentrate, d‐a‐Tocopheryl polyethylene glycol 1000 succinate (TPGS), and polysorbate 40 are mixed (Table 1). The mixture is heated up to 90°C ± 2°C to melt and stirred (500 rpm) until a homogeneous, clear mixture is obtained. The needed amount of drug is added to the lipid mixture and is stirred (500 rpm) until it fully dissolves. The needed amount of deionized water with the NaCl dissolved in it is heated up to 90°C ± 2°C, and it is added dropwise to the homogeneous mixture obtained under stirring. The obtained dispersion is cooled down under stirring (300 rpm) to 20°C ± 2°C to give the nanoparticle dispersion. The detailed formulation, composition, and processing parameters are summarized in Table S12.

### 4.3 Purification and Dialysis of Samples

(a) The procedure for purification of the prepared LNPs involves the removal of the residual amount of surfactant used in the preparation of LNPs and is described as “washed from surfactants” in Table 2. A volume of 20 mL of the tested dispersion was filtered through a 0.45 µm glass fibre syringe filter and introduced into a Spectrum Spectra/Por 7 dialysis tubing with a 50 kD MWCO. A permeable membrane allowed surfactants to pass through, while restricting particle movement. It was sealed at both ends and immersed in a 5‐litre glass beaker with 4500 mL of 0.9% NaCl solution in MilliQ ultrapure water at 25°C for 48 h with continuous stirring, replacing the washing solution every 12 h. After 48 h, ten 5 mL samples of the washing solution were scanned in the UV region from 200 nm to 400 nm, using 0.9% NaCl in MilliQ ultrapure water as a blank. The signal was analyzed to determine the lack of measurable value (X_L_) of solutes in the final washing solution. The calculation of X_L_ is expressed by the equation:

(1) XL=Xbi+k·Sbi

With X_bi,_ the mean of the blank measurements, which serves as a baseline, S_bi_ is the standard deviation of the blank measurements, indicating measurement variability; k is a numerical factor chosen according to the desired confidence level for detection. In our tests, k = 3.

The equation sets a limit of detection, ensuring reliable analytical results and aiding decision‐making for an extra 12‐h washing period(s) based on analyte quantities in the samples.

Dialysis of the samples continued until the absence of an absorbance peak corresponding to the surfactant used. All samples prepared were packed in aseptic conditions in a Class 2A biological safety cabinet (Biobase Class IIA Biological Safety Cabinet).

(b) Dialysis was carried out against MilliQ ultrapure water for 24 h, replacing the washing solution every 6 h at 200 rpm. Spectra/Por Float‐A‐Lyzer G2 Volume 1 mL, 8–10 kDa MWCO membrane was used for dialysis. The dialyzed samples were used for batch‐mode dynamic light scattering (DLS) and small‐angle x‐ray scattering (SAXS).

### 4.4 Characterization of Lipid Nanoparticles

#### 4.4.1 Dynamic Light Scattering (DLS)

Batch‐mode DLS studies were carried out on a Zetasizer Nanoseries (Malvern Instruments, UK), equipped with Zetasizer software (version 8.02). Data were collected at a scattering angle of 173° and with a laser wavelength of λ = 632 nm. Data evaluation was carried out by using Zetasizer Software (version 8.02). Measurements were performed in an aqueous solution of 0.9% NaCl or MilliQ ultrapure water using disposable cuvettes (ZEN0040). The automatic attenuator factor was between 6 and 7. Measurements were performed at 25°C, all samples were equilibrated for 5 min before each measurement, and the software automatically selected the acquisition time for each measurement according to the sample conditions. The mean particle size and size distribution were assessed by intensity (intensity‐PSD). All measurements were performed in a minimum of triplicate.

#### 4.4.2 Zeta Potential

The zeta potential studies were carried out on a Zetasizer Nanoseries (Malvern Instruments, UK), equipped with Zetasizer software (version 8.02). Data evaluation was carried out by using Zetasizer Software (version 8.02). Measurements were performed in an aqueous solution of 1 mm NaCl using disposable folded capillary cells (DTS1070). Measurements were performed at 25°C, and all samples were equilibrated for 5 min before each measurement. The results were determined by the mean of five measurements. Between each measurement, there was a 300‐s delay. The model for the F(ka) selection was Smoluchowski (1.5). The measurement setting was selected with automatic attention and automatic voltage. The analysis mode was monomodal unless otherwise stated.

#### 4.4.3 Asymmetric Flow Field‐Flow Fractionation (AF4)

AF4 measurements were performed with an Eclipse Neon Asymmetric flow field‐flow fractionation (AF4) system (Wyatt Technologies Corp., Germany). The system is equipped with a DAWN Neon MALS (18 angles, operating at a wavelength of 659 nm) with online QELS, OptiLab dRI (operating at a wavelength of 658 nm) (Wyatt Technologies Corp., Germany), and Agilent 1260 Infinity II MWD, an Agilent Infinity II isocratic pump with an integrated degassing system and an autosampler (Agilent Technologies 1260 Infinity series, Agilent Technologies, USA) (AF4‐UV‐QELS‐MALS‐dRI).

Measurements were carried out in a carrier liquid of 10 mm PBS buffer at pH 7.4, containing 200 mg L^−1^ NaN_3_. An Eclipse short channel with DCM and a defined channel height of 350 µm asymmetric channel spacer was connected. A regenerated cellulose (RC) ultrafiltration membrane (Wyatt Technologies Corp., Germany) with a molecular weight cut‐off (MWCO) of 10 kDa was used. The samples were injected into the AF4 separation channel using the autosampler. The AF4 method was optimized to achieve optimal fractionation of the lipid nanoparticles (see Figure S1 and Table S5). The data was collected by the Astra 8.1.2 software (Wyatt Technologies Corp., Germany). The following method was applied: Detector flow rate was set to 0.5 mL min^−1^, for the separation of an isocratic step with a V_x_ of 3 mL min^−1^ for 12 min (Elution; Focus; Focus Inject; Elution), followed by an exponential V_x_ gradient (slope 8) from 3 mL min^−1^ to 0.05 mL min^−1^ within 25 min. The last steps consisted of an isocratic V_x_ of 0.05 mL min^−1^ for 5 min, followed by an isocratic V_x_ of 0 mL min^−1^ for 5 min. The MWD lamp was set to 250 nm, 280 nm, 300 nm, 310 nm, and 330 nm. Three injections of 25 µL and one of 250 µL were performed for each sample, and the injection flow rate was set to 0.2 mL min^−1^. Method performance was validated through recovery measurement using UV detection (280 nm). Recovery was evaluated by comparing the peak areas of the full separation method and the focus‐flow inject analysis (FIA) against an FIA baseline (100% recovery) (Table S6). Precision of retention time and hydrodynamic radius were also evaluated (Tables S7 and S8).

For the indirect determination and quantification of the non‐encapsulated drug present with in a LNP formulation, using AF4 with the cross‐flow coupled to the UV–vis detector, the following method was applied (Figure S13 and Table S10): Detector flow rate was set to 0.3 mL min^−1^, for the separation of an isocratic step with a V_x_ of 1 mL min^−1^ for 36 min (Elution; Focus; Focus Inject; Elution), followed by an isocratic step with a V_x_ of 1 mL min^−1^ for 10 min exponential after which the V_x_ gradient decreased from 1 to 0.05 mL min^−1^ within 10 min. The last steps consisted of an isocratic V_x_ of 0.05 mL min^−1^ for 5 min, followed by an isocratic V_x_ of 0 mL min^−1^ for 5 min. The MWD lamp was set to 250 nm. Three injections of 25 were performed for each sample, and the injection flow rate was set to 0.2 mL min^−1^. For quantification of free drug in the cross‐flow, a calibration curve was established at 250 nm by injections of quinine standards over the range of 0.05–0.21 mg mL^−1^, yielding a linear relationship (R^2^ = 0.9983) between peak area and concentration. The limit of detection (LOD) and limit of quantification (LOQ) were calculated as 3.3 σ/S and 10 σ /S, respectively, where σ is the standard deviation of the detector response across the linear range, and S is the slope of the calibration. The LOD = 0.0050 mg mL^−1^ and LOQ = 0.0152 mg mL^−1^ (Table S11).

For the collection of size‐dependent fluorescence spectra, an FLD Infinity II 1260 (Agilent Technologies) was included in the setup. The excitation wavelengths were set to 250 nm and 350 nm. The emission spectra were recorded from 250 nm to 600 nm during a time of 10–40 min of elution using the separation protocol as described above.

#### 4.4.4 Fluorescence in Batch

Fluorescence spectra were recorded using an FS5 Spectrofluorometer (Edinburgh Instruments Corp., USA), equipped with a xenon flash lamp. LNP samples were prepared by dispersing 10 µL of the LNP suspension in 990 µL of 0.9% NaCl. Excitation was set at 288 nm, and emission spectra were collected from 300 nm to 600 nm. The slit widths were set to 3 nm. Measurements were performed at room temperature.

#### 4.4.5 Transmission Electron Microscopy (TEM)

Cryo‐transmission electron microscopy (cryo‐TEM) images were recorded in Libra 120 microscope (Carl Zeiss Microscopy Deutschland GmbH, Oberkochen, Germany). 2 µL to 4 µL of specimen was placed onto a holey carbon TEM grid (Quantifoil R3.5/1, 300 mesh), blotted with filter paper for 0.2 s to 1 s and vitrified in liquid ethane at ‐178°C using a Grid Plunger (Leica Microsystems GmbH, Wetzlar, Germany). Frozen grids were transferred into a Gatan 626 (Gatan GmbH, München, Germany) cryo‐TEM holder. Images were recorded at an accelerating voltage of 120 kV while keeping the specimen at −170°C. To study the size distribution of particles, ImageJ software (version) was used. To quantify the size distribution, 30–40 spherical particles and 10–20 elongated particles were manually measured in ImageJ, from three different images, to obtain the average size distribution. The raw data were processed in Origin Pro 2021 and plotted using a Gaussian distribution curve.

#### 4.4.6 X‐ray Scattering (SAXS and WAXS)

Small‐angle X‐ray scattering (SAXS) and wide‐angle X‐ray scattering (WAXS) measurements were performed on a Ganesha 300 XL+ (SAXLAB) in vacuum. The instrument is equipped with a pinhole‐focused X‐ray beam (αCuK_α_, λ = 1.542 Å) and a Pilatus 300k pixel detector (pixel size: 172 × 172 µm^2^). SAXS and WAXS data were recorded at a sample detector distance of 1052 mm and 110 mm, respectively. Quartz glass capillaries were loaded with lipid nanoparticle solutions at different concentrations (based on lipid fraction) of 11 mg mL^−1^, 4 mg mL^−1^, 2 mg mL^−1^, 0.5 mg mL^−1^ in pure water or 0.9% NaCl. SAXS integration times were chosen between 2 h and 4 h, while WAXS data was only obtained for the 11 mg mL^−1^ concentration, integrating for 30 min for each sample. 2D data were azimuthally averaged to obtain 1D scattering curves, after standard data correction procedures (e.g. dark current subtraction, flat field correction). SAXS data were normalized and converted to absolute intensity using water as a standard and then referenced to the respective pure solution without the sample. SAXS data for LNP that consist only of a carnauba wax core (LNP_1_‐CW‐TPGS) and LNP_5_ in water were modelled in SasView version. 5.0.5 [44] using the core‐shell bicelle model. X‐ray CuK_α_ scattering length density (SLD) of water was calculated using the NIST tool [45] to SLD_H2O_ = 9.47 · 10^−6^ Å^−2^. WAXS data were scaled to the same background intensity level (amorphous SiO_2_ background) and then corrected for the background using a spline function.

### 4.5 Biocompatibility Studies

#### 4.5.1 Human Samples

Human skin samples were obtained from clinically healthy donors using surplus tissue collected during elective surgical procedures (breast and abdominal areas) at the Department of Orthopaedics, Trauma Surgery, and Plastic Surgery, University Hospital Leipzig. In addition, peripheral blood samples were collected from healthy adult volunteers. All human samples were collected following written informed consent and with prior approval from the Ethics Committee of Leipzig University (approval number EK092‐20), in accordance with the principles of the Declaration of Helsinki.

#### 4.5.2 Isolation and Culture of Primary Human Macrophages and Fibroblasts

Human macrophages were generated as previously described [46]. Briefly, peripheral blood mononuclear cells (PBMCs) were isolated from human blood by density‐gradient centrifugation using Ficoll‐Paque PLUS (Cytiva Sweden AB). Monocytes were then enriched from PBMCs to a purity of >95% using the CD14+ Cell Isolation Kit (Miltenyi). Enriched monocytes were seeded at a density of 4 × 10^5^ cells/ml in RPMI‐1640 medium (Anprotec) supplemented with 1% (v/v) PenStrep (Anprotec), as well as 10% (v/v) heat‐inactivated fetal calf serum (FCS, Anprotec) to complete RPMI medium. Monocyte‐to‐macrophage differentiation was induced by the addition of 50 ng/mL macrophage colony‐stimulating factor (M‐CSF, Miltenyi) and continued culture for six days under standard culture conditions.

Primary dermal fibroblasts were isolated from human skin tissue as previously described [46]. Briefly, epidermal layers were separated from the dermis using Dispase II (Roche Diagnostics), followed by enzymatic digestion of the dermal compartment with collagenase (Sigma‐Aldrich) to release fibroblasts. The resulting cell suspension was filtered through a 70 µm cell strainer (Greiner Bio‐One) to eliminate residual tissue fragments. Fibroblasts were cultured in Dulbecco's Modified Eagle Medium (DMEM; Anprotec) supplemented with 1% (v/v) PenStrep (Anprotec) and 10% (v/v) FCS to complete DMEM. Fibroblasts were grown to 80% confluence, and only cells between passages 2 and 4 were used for downstream experiments.

Macrophages and fibroblasts were maintained at 37°C in a humidified atmosphere containing 5% CO_2_ for the duration of all experiments.

#### 4.5.3 LNP Stimulation of Macrophages and Fibroblasts

Macrophages were seeded at a density of 80 000 cells per well in 48‐well plates using 250 µL of complete RPMI‐1640 medium. Cells were then stimulated with 250 µL of various LNP formulations, prepared at the indicated concentrations in complete RPMI‐1640 medium. For macrophage activation, cells were additionally stimulated with 100 ng mL^−1^ lipopolysaccharide (LPS, Sigma), as described previously [47]. Fibroblasts were seeded at 20 000 cells per well in 24‐well plates in 500 µL of complete DMEM and stimulated with 500 µL of LNP formulations prepared at different concentrations in complete DMEM. For cell viability assays, macrophages and fibroblasts were seeded in 96‐well plates at 15 000 and 2 000 cells per well, respectively, in 100 µL of their respective complete media. After cell attachment, 100 µL of the corresponding LNP formulations were added to each well.

#### 4.5.4 Ex Vivo Human Skin Model

The ex vivo skin culture model was established based on the method described by Wilkinson et al. [48]. Human skin samples were obtained from surgical procedures and immediately transferred to the laboratory in holding medium composed of DMEM+GlutaMAX (Gibco) supplemented with 1% (v/v) PenStrep (Anprotec), 1% (v/v) L‐Glutamine (Gibco), and 2.5 µg mL^−1^ Amphotericin (CHEPLA PHARM). All subsequent handling was performed promptly under sterile conditions in a laminar flow cabinet. To remove residual blood, skin samples were washed three times with Hank's Balanced Salt Solution (HBSS, Gibco) containing 4% (v/v) antibiotic‐antimycotic solution, followed by a final wash in PBS (Anprotec). The tissue was then cut into small squares (approximately 8 × 8 mm^2^) and transferred into a pre‐prepared 12‐well plate (see Figure S25A). Each well contained two sterile absorbent pads overlaid with a sterile nylon membrane (Merck) pre‐soaked in skin culture medium (DMEM+GlutaMAX (Gibco), supplemented with 1% (v/v) PenStrep (Anprotec), 1% (v/v) L‐Glutamine (Gibco), 2.5 µg mL^−1^ Amphotericin (CHEPLA PHARM), 100 U/mL insulin (Merck), and 1 µm dexamethasone (Sigma). One skin piece was placed epidermis side up on each membrane. Each well was filled with skin medium to enable air‐liquid interface culture. Skin samples were incubated at 37°C in a humidified atmosphere with 5% CO_2_ for up to 14 days. The culture medium was refreshed every 2–3 days.

#### 4.5.5 LNP Injection into Ex Vivo Skin

For LNP administration, skin pieces were placed epidermis‐side up on a sterile filter (Figure 6A). A total volume of 100 µL of LNP formulations, diluted at the indicated concentrations in PBS, was carefully injected at the dermal–hypodermal interface. After injection, the skin was carefully washed in PBS before being placed back onto the nylon membrane in the culture dish. Sham injections with 100 µL PBS served as treatment controls, and untreated skin served as an additional control. Following injection, all skin samples were cultured at 37°C in a humidified incubator with 5% CO_2_ for the specified time points. Culture medium was replaced every 2–3 days.

### 4.6 Sample Analysis

#### 4.6.1 Assessment of Cell Viability

Cell viability of macrophages and fibroblasts was assessed using the Cell Proliferation Kit II (XTT; Roche Diagnostics), following the manufacturer's protocol. The assay is based on the metabolic activity of viable cells, which reduces the XTT reagent to a water‐soluble formazan dye. The resulting color intensity, measured spectrophotometrically, correlates directly with the number of metabolically active cells. Viability was expressed as the ratio of the change in optical density (ΔOD) relative to untreated control cells cultured without LNP exposure.

#### 4.6.2 Real‐Time Monitoring of Fibroblast Proliferation

Live‐cell proliferation of fibroblasts was monitored using the Incucyte S3 live‐cell imaging system (Sartorius & Essen, BioScience). Cells were seeded in 24‐well plates and stimulated with LNPs as described in the LNP Stimulation of Macrophages and Fibroblasts Section 4.5.3. Proliferation was tracked over 72 h, with 12 images captured per well at 6‐h intervals. Cell confluence was quantified using Incucyte analysis software (Sartorius), which calculated changes in confluence over time based on the acquired images for each condition.

#### 4.6.3 RNA Isolation and Quantitative Real‐Time PCR

For cell culture experiments, total RNA was isolated directly from adherent macrophages and fibroblasts using the RNeasy Mini Kit (Qiagen), following the manufacturer's instructions. In skin culture experiments, the subcutaneous fat layer was first carefully separated from the skin, and the dermis and epidermis were subsequently separated from the remaining skin tissue by digestion with dispase for 2 h. Isolated fat and dermal tissues were homogenized, and total RNA was extracted using the ReliaPrep RNA Tissue Miniprep System (Promega), according to the manufacturer's protocol. RNA concentration and purity were assessed using a NanoDrop ND‐1000 spectrophotometer (NanoDrop Technologies, Wilmington, DE, USA). For cDNA synthesis, 250 ng of total RNA was reverse transcribed using the LunaScript RT SuperMix Kit (New England Biolabs), following the manufacturer's guidelines. Quantitative real‐time PCR was performed using Luna Universal qPCR Master Mix (New England Biolabs) with gene‐specific primers (listed in Table S13). All PCR products were designed to span intron‐exon boundaries.

Gene expression levels were quantified using standard curves generated from cloned cDNA and normalized to RPS26 (for cell samples) or GAPDH (for skin‐derived dermis and fat).

#### 4.6.4 Cytokine Quantification

Concentrations of human TNF, IL‐1β, MCP‐1, and IL‐10 in cell‐free supernatants from macrophage cultures were measured using corresponding ELISA Kits (Thermo Fisher Scientific), according to the manufacturer's protocols.

#### 4.6.5 Histology and Lipid and Adipocyte Staining of Skin Samples

Skin samples were cryo‐frozen (Tissue Freezing Medium, Leica) and paraffin‐embedded and skin sections were prepared for immunohistochemistry or immunofluorescence staining.

For morphometric analysis, paraffin‐embedded skin tissue sections (5 µm) were deparaffinized, rehydrated, and stained with hematoxylin and eosin using standard protocols. Stained sections were dehydrated, mounted with coverslips, and imaged by brightfield microscopy.

Oil Red O and LipidTOX staining were performed on cryosections of frozen skin tissue (50 µm thick). Sections were fixed in 4% paraformaldehyde, rinsed with distilled water, and incubated either with freshly prepared Oil Red O working solution (0.3% Oil Red O in 60% isopropanol, Sigma‐Aldrich) for 20 min or with HCS LipidTOX Neutral Lipid Stain (Invitrogen), diluted 1:1000 in PBS, for 30 min at room temperature. Following staining, sections were rinsed with PBS. For LipidTOX‐stained samples, nuclei were counterstained with DAPI (1:500 in PBS) for 10 min at room temperature. After a final wash, slides were mounted and imaged using brightfield microscopy (Oil Red O) or fluorescence microscopy (LipidTOX).

Perilipin (PLIN‐1) staining was performed on paraffin‐embedded skin tissue sections (10 µm thick). Following deparaffinization, antigen retrieval was carried out in citrate buffer (pH 6.0) for 30 min. After blocking and washing, sections were incubated overnight at 4°C with anti‐PLIN‐1 primary antibody (Abcam, 1:500 dilution). The next day, sections were incubated with Alexa Fluor 546‐conjugated secondary antibody (Invitrogen, 1:500), followed by nuclear counterstaining with DAPI (Invitrogen, 1:500).

Microscopy of all samples was performed with a Keyence BZ‐X800 microscope (Biorevo) equipped with a fluorescence and image‐capturing system.

#### 4.6.6 TUNEL Assay

TUNEL staining was performed on paraffin‐embedded tissue sections (5 µm) using the ApopTag Fluorescein In Situ Apoptosis Detection Kit (Merck), following the manufacturer's protocol. Sections were deparaffinized, rehydrated, and processed according to kit instructions. Nuclei were counterstained with DAPI (Invitrogen, 1:500), and fluorescence imaging was carried out using a Keyence BZ‐X800 microscope (Biorevo).

#### 4.6.7 Quantification of LNP Penetration through Skin

To assess the penetration of LNPs through skin, samples injected with fluorescein‐loaded LNP_1_‐FL were placed in transwell inserts (translucent, 0.4 µm pore size; Greiner Bio‐One) within a 12‐well plate. Both the lower chamber and the transwell insert were filled with a fixed volume of skin culture medium, allowing for air‐liquid interface conditions. At the indicated time points, medium from the lower chamber was collected and replaced with the same volume of fresh medium. Fluorescence in the collected medium was quantified using a fluorescence plate reader (SinergyHT, BioTek).

### 4.7 Statistical Analysis

Comparisons involving more than two groups over time were analyzed using two‐way ANOVA followed by Tukey's multiple comparisons test. Statistical calculations were performed using GraphPad Prism, version 10. *p*‐Values ≤ 0.05 were considered statistically significant. Levels of significance are indicated as follows: ^*^ *p* < 0.05; ^**^ *p* < 0.01; ^***^ *p* < 0.001; ^****^ *p* < 0.0001.

## Author Contributions

The manuscript was written through the contributions of all authors. All authors have approved the final version of the manuscript.

## Funding

The studies were performed within the 3D4D2 project carried out under the M‐ERA.NET 2 scheme (European Union's Horizon 2020 research and innovation programme, grant No. 685451) and co‐funded by the Saxon State Ministry for Science, Culture and Tourism (Germany), grant No. 100579959, as well as from the tax funds based on the budget passed by the Saxon state parliament.

## Conflicts of Interest

The authors declare no conflict of interest.

## Supporting information




**Supporting File**: smll73101‐sup‐0001‐SuppMat.pdf.

## Data Availability

The data that support the findings of this study are available from the corresponding author upon reasonable request.
