# Supplementary material for: Disc‐Toroid Hybrid Lipid Nanoparticles for Efficient Drug Encapsulation and Subcutaneous Delivery
Source: Small. 2026 Mar 12;22(26):e00052. doi: 10.1002/smll.202600052 (PMC13155039; doi:10.1002/smll.202600052)
Supplement: Supplementary file 1 — Supporting File: smll73101‐sup‐0001‐SuppMat.pdf. [file SMLL-22-e00052-s001.pdf]

Supplementary Information for

Disc-Toroid Hybrid Lipid Nanoparticles for Efficient Drug Encapsulation and Subcutaneous Delivery

Zanelle van Niekerk‡, Rima Nuwayhid‡, Stefaniya Gaydarova, Eva Bittrich, Natalia Makarova†, Susanne Boye, Christo Tzachev, Jan C. Simon, Sandra Franz‡, Albena Lederer‡**

Zanelle van Niekerk, Albena Lederer

Stellenbosch University, Department of Chemistry and Polymer Science, Private Bag X1, Matieland, 7599, South Africa

Email: alederer@sun.ac.za

Zanelle van Niekerk, Eva Bittrich, Natalia Makarova, Susanne Boye, Albena Lederer

Leibniz-Institut für Polymerforschung Dresden e.V., Hohe Strasse 6, 01069 Dresden, Germany

† Present Address: Swabian Instruments, Stammheimer Str. 41, 70435 Stuttgart, Germany

Email: lederer@ipfdd.de

Stefaniya Gaydarova, Christo Tzachev

Faculty of Chemistry and Pharmacy, Sofia University "St. Kliment Ohridski", 1 J. Bourchier Blvd., 1164 Sofia, Bulgaria

Jan C. Simon, Sandra Franz

Department of Dermatology, Venereology and Allergology, University of Leipzig, Medical Faculty, Leipzig, Germany

Email: sandra.franz@medizin.uni-leipzig.de

Rima Nuwayhid

Clinic for Orthopaedics, Trauma Surgery and Plastic Surgery, Leipzig University Hospital, Leipzig, Germany

‡These authors contributed equally

Size and stability characterization of LNPs using batch-DLS

Table S1: Size and polydispersity index (PDI) of the various LNP formulations as determined by DLS over time

Sample	Aqueous media	Time (months)	Cumulants Method		PSD analysis (Intensity)
			z-average (d.nm)[a]	PDI[a]	Size (d.nm)[a, b]
LNP_5	0.9% NaCl	t_0	39.4 ± 0.2	0.171 ± 0.007	43.5 ± 1.7
		t_5	36.2 ± 0.1	0.090 ± 0.010	39.9 ± 0.4
LNP_5-Q_{10}	0.9% NaCl	t_0	35.3 ± 0.0	0.065 ± 0.003	37.9 ± 0.0
		t_5	35.3 ± 0.1	0.058 ± 0.007	37.9 ± 0.2
LNP_5-DHA_3	0.9% NaCl	t_0	35.3 ± 0.1	0.087 ± 0.021	38.9 ± 1.0
		t_5	35.8 ± 0.1	0.103 ± 0.010	39.8 ± 0.3
LNP_1	0.9% NaCl	t_0	37.6 ± 0.2	0.116 ± 0.005	42.2 ± 0.3
		t_{18}	38.1 ± 0.1	0.126 ± 0.006	42.2 ± 0.8
LNP_1-Q_{10}	0.9% NaCl	t_0	33.9 ± 0.1	0.082 ± 0.007	37.1 ± 0.4
		t_{18}	35.7 ± 0.3	0.151 ± 0.017	38.9 ± 0.5
LNP_1-W	ultrapure water	t_0	32.8 ± 0.1	0.081 ± 0.004	35.9 ± 0.3
		t_{18}	33.1 ± 0.2	0.148 ± 0.012	37.4 ± 1.0
LNP_1-CW-TPGS	ultrapure water	t_0	35.0 ± 0.1	0.145 ± 0.012	40.2 ± 0.6
		t_{18}	35.4 ± 0.4	0.175 ± 0.011	40.3 ± 1.0
LNP_1-CW-T40	ultrapure water	t_0	48.2 ± 0.2	0.167 ± 0.006	58.5 ± 0.8
		t_{18}	48.5 ± 0.1	0.164 ± 0.006	58.8 ± 0.5
LNP_1-TPGS	ultrapure water	t_0	38.4 ± 0.1	0.193 ± 0.008	46.0 ± 1.2
		t_{18}	39.6 ± 0.2	0.197 ± 0.005	45.9 ± 1.7
LNP_1-T40	ultrapure water	t_0	39.3 ± 0.1	0.133 ± 0.006	45.8 ± 0.4
		t_{18}	40.3 ± 0.2	0.128 ± 0.013	46.2 ± 0.8
LNP_1-FL	0.9% NaCl	-	39.4 ± 0.3	0.176 ± 0.005	43.1 ± 1.1

(a) The average and mean standard deviation of the z-average, polydispersity index (PDI), and particle size (d.nm) are calculated from 3-5 replicates. b) The particle size (d.nm) is intensity-based.

Table S2: Batch-to-batch comparison of LNP formulation by DLS

Sample	Aqueous media	Cumulants Method		PSD analysis (Intensity)
		z-average (d.nm)[a]	PDI[a]	Size (d.nm)[a,b]
LNP_5	0.9% NaCl	39.4 ± 0.2	0.171 ± 0.007	43.5 ± 1.7
LNP_5-WS	0.9% NaCl	35.8 ± 0.5	0.085 ± 0.014	39.3 ± 0.3
LNP_5-B2	0.9% NaCl	36.4 ± 0.2	0.083 ± 0.010	39.7 ± 0.3
LNP_5-B2-WS	0.9% NaCl	35.1 ± 0.0	0.034 ± 0.011	37.1 ± 0.2
LNP_5-B3	0.9% NaCl	35.7 ± 0.2	0.089 ± 0.010	39.1 ± 0.1
LNP_5-B3-WS	0.9% NaCl	34.7 ± 0.1	0.042 ± 0.009	36.7 ± 0.1
LNP_5-B4	0.9% NaCl	35.6 ± 0.2	0.085 ± 0.007	38.9 ± 0.4
LNP_5-B4-WS	0.9% NaCl	34.9 ± 0.1	0.063 ± 0.007	37.5 ± 0.1
LNP_5-Q_{10}	0.9% NaCl	35.3 ± 0.0	0.065 ± 0.003	37.9 ± 0.0
LNP_5-B2-Q_{10}	0.9% NaCl	32.9 ± 0.2	0.044 ± 0.017	34.9 ± 0.3
LNP_5-B4-Q_{20}	0.9% NaCl	34.2 ± 0.1	0.084 ± 0.009	37.4 ± 0.3
LNP_5-DHA_3	0.9% NaCl	35.3 ± 0.1	0.087 ± 0.021	38.9 ± 1.0
LNP_5-B2-DHA_5	0.9% NaCl	34.0 ± 0.1	0.047 ± 0.009	36.2 ± 0.2
LNP_5-B4-DHA_3	0.9% NaCl	35.3 ± 0.1	0.062 ± 0.014	37.9 ± 0.4

*(a) The average and mean standard deviation of the z-average, polydispersity index (PDI), and particle size (d.nm) are calculated from 3-5 replicates. (b) The particle size (d.nm) is intensity-based. *Batch-to-batch reproducibility (coefficient of variation, CV%) was calculated using the z-average means of independent formulations with identical composition. CV% values: LNP_5 = 4.86% (n =4), LNP_5-WS = 1.36 % (n=4), LNP_5-Q_{10} = 4.98 % (n=2), LNP_5-DHA_3 = 0.00 % (n=2)*

Table S3: Zeta (ζ) potential values for the various LNP formulations

Sample	Aqueous media	Zeta potential (mV)[a]	Conductivity (mS/cm)
LNP_5	1.5mM NaCl	-8.90 ± 0.57	0.146 ± 0.001
LNP_5-Q_{10}	1.5 mM NaCl	-15.10 ± 0.75	0.157 ± 0.001
LNP_5-DHA_3	1.5mM NaCl	-9.27 ± 0.37	0.150 ± 0.001
LNP_1	1.5 mM NaCl	-7.41 ± 1.59	0.229 ± 0.001
LNP_1-Q_{10}	1.5 mM NaCl	-12.50 ± 0.94	0.215 ± 0.000
LNP_1-W	1 mM NaCl	-8.41 ± 0.54	0.141 ± 0.001
LNP_1-CW-TPGS	1 mM NaCl	-10.90 ± 0.61	0.150 ± 0.001
LNP_1-CW-T40	1 mM NaCl	-13.80 ± 0.35	0.151 ± 0.000
LNP_1-TPGS	1 mM NaCl	-11.50 ± 1.30	0.150 ± 0.000
LNP_1-T40	1 mM NaCl	-11.70 ± 0.55	0.151 ± 0.000
LNP_1-FL	1 mM NaCl	-11.40 ± 1.44	0.217 ± 0.001

(a) Zeta potential measurements were performed at 25°C, with a monomodal analysis function.

Table S4: Size and polydispersity index of different sample concentrations of LNP_1-CW-TPGS as determined by batch-mode DLS for SAXS measurements

Sample	Concentration[a] (mg mL^{-1})	Cumulants Method		PSD analysis (Intensity)
		z-average (d.nm)[b]	PDI[b]	Size (d.nm)[b,c]
LNP_1-CW-TPGS [d]	11.02	29.9 ± 0.0	0.166 ± 0.006	34.4 ± 0.7
	4.80	31.5 ± 0.1	0.137 ± 0.005	36.5 ± 0.5
	2.42	31.6 ± 0.2	0.149 ± 0.010	36.6 ± 0.9
	0.48	35.4 ± 0.2	0.146 ± 0.011	40.0 ± 1.1

(a) Concentration based on lipid fraction. (b) The average and mean standard deviation of the z-average, polydispersity index (PDI), and particle size (d.nm) are calculated from 3-5 replicates. (c) The particle size (d.nm) is intensity-based. d) Aqueous media: MilliQ ultrapure water.

Characterization of LNP using AF4-MD

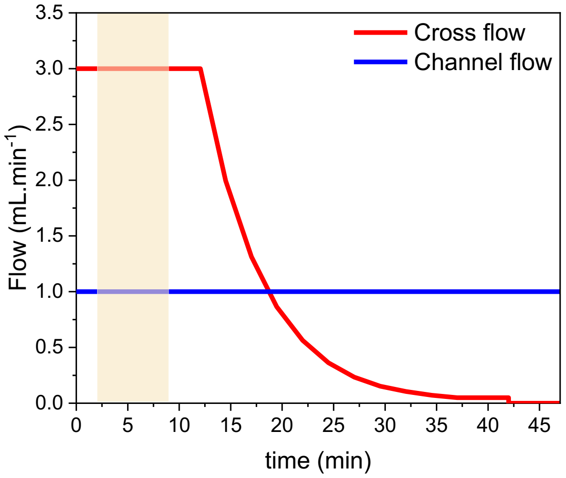

Figure S1. AF4 separation flow profile for LNP using 10 mM PBS at pH 7.4 as eluent.

Table S5: Experimental conditions for the characterization of LNP by AF4-MD

**Conditions**	
Injection volume:	25 µL and 250 µL (QELS)
Spacer:	350 µm
Membrane:	10 kDa (regenerate cellulose, Wyatt)
Detector flow:	0.5 mL min^{-1}
Eluent:	10 mM PBS buffer at pH 7.4, containing 200 mg L^{-1} NaN_3
Channel:	Eclipse short channel with DCM, Wyatt Technologies Corp.
**Detector**	
Agilent 1260 Infinity II MWD	Wavelength: 250 nm, 280 nm, 300 nm, 310 nm, and 330 nm
Multi-angle light scattering (MALS) with online QELS (DAWN Neon MALLS, Wyatt Technologies Corp.)	18 angles, operating at a wavelength of 659 nm
OptiLab dRI	Operating at a wavelength of 658 nm
Agilent FLD Infinity II 1260	Excitation wavelengths: 250 and 350 nm, emission spectra 250 – 600 nm with 5 nm step size, photomultiplier tube (PMT) gain 12M, intensities are normalized to I_{max} of loaded LNP_1-Q_{10}
Method:	Isocratic step with a V_x of 3 mL min^{-1} for 12 min (Elution; Focus; Focus Inject; Focus, Elution). Exponential V_x gradient (slope 8) from 3 to 0.05 mL min^{-1} within 25 min was used. Isocratic V_x of 0.05 mL min^{-1} for 5 min. Isocratic V_x of 0 mL min^{-1} for 5 min. Injection flow rate: 0.2 mL min^{-1}

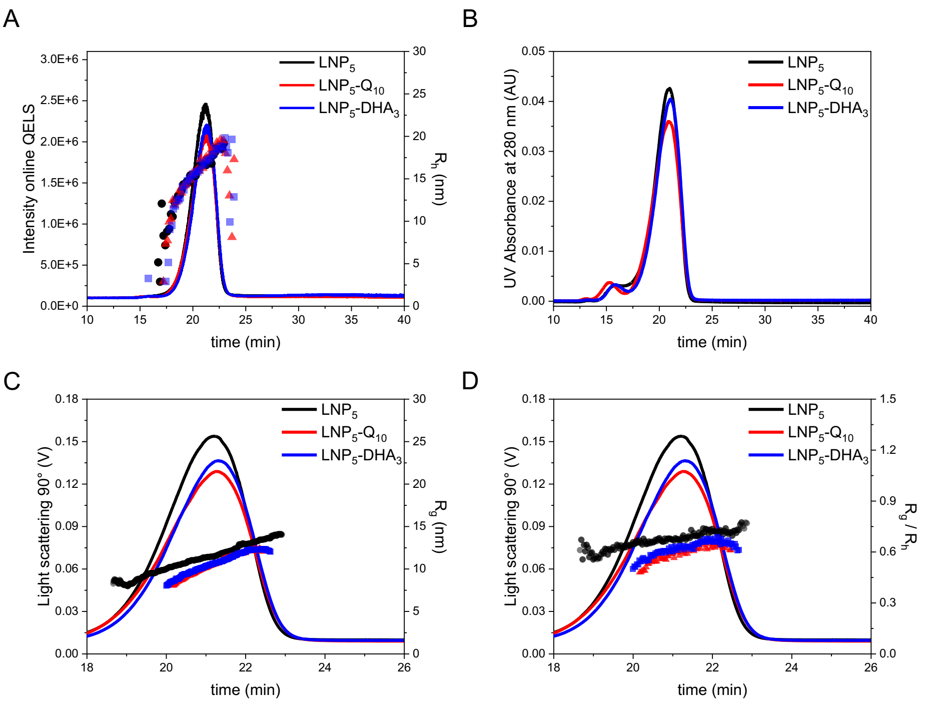

Figure S2. AF4 separation of LNP$_5$, LNP$_5$-Q_{10}, and LNP$_5$-DHA$_3$. **(A)** Overlay of online QELS intensity vs. time, highlighting the R_h distribution across the eluting peak. **(B)** Overlay of UV absorbance at 280 nm vs. time. **(C)** Overlay of light scattering signal vs. elution time, highlighting the R_g distribution across the eluting peak. **(D)** Overlay of light scattering signal vs. elution time with R_g/R_h shape factor across the peak.

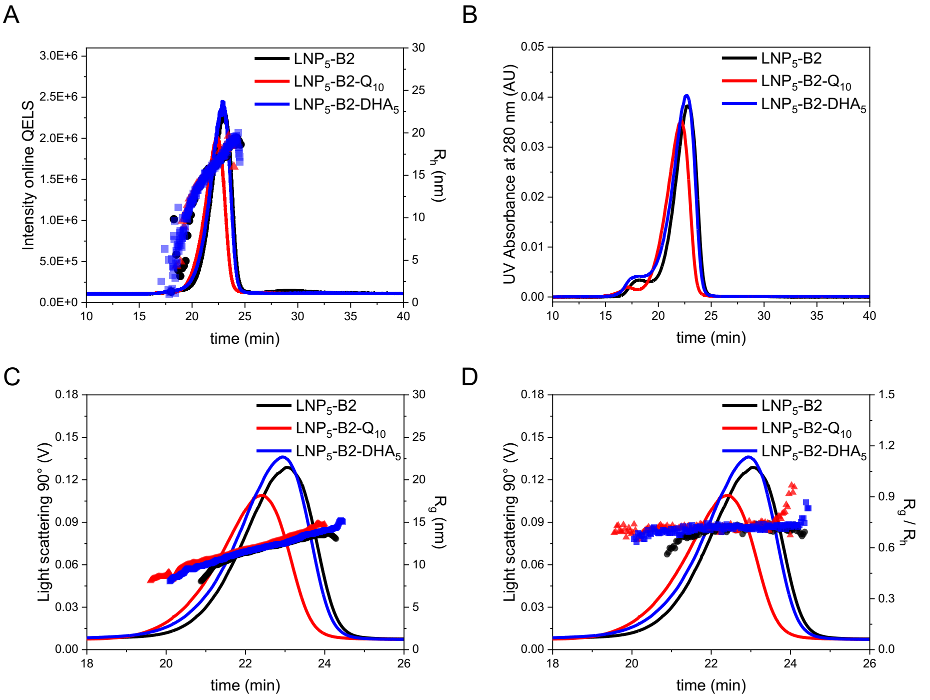

Figure S3. AF4 separation of LNP$_5$-B2, LNP$_5$-B2-Q_{10}, and LNP$_5$-B2-DHA$_5$ **(A)** Overlay of online QELS intensity vs. time, highlighting the R_h distribution across the eluting peak. **(B)** Overlay of UV absorbance at 280 nm vs. time. **(C)** Overlay of light scattering signal vs. time, highlighting the R_g distribution across the eluting peak. **(D)** Overlay of light scattering signal vs. elution time with R_g/R_h shape factor across the peak.

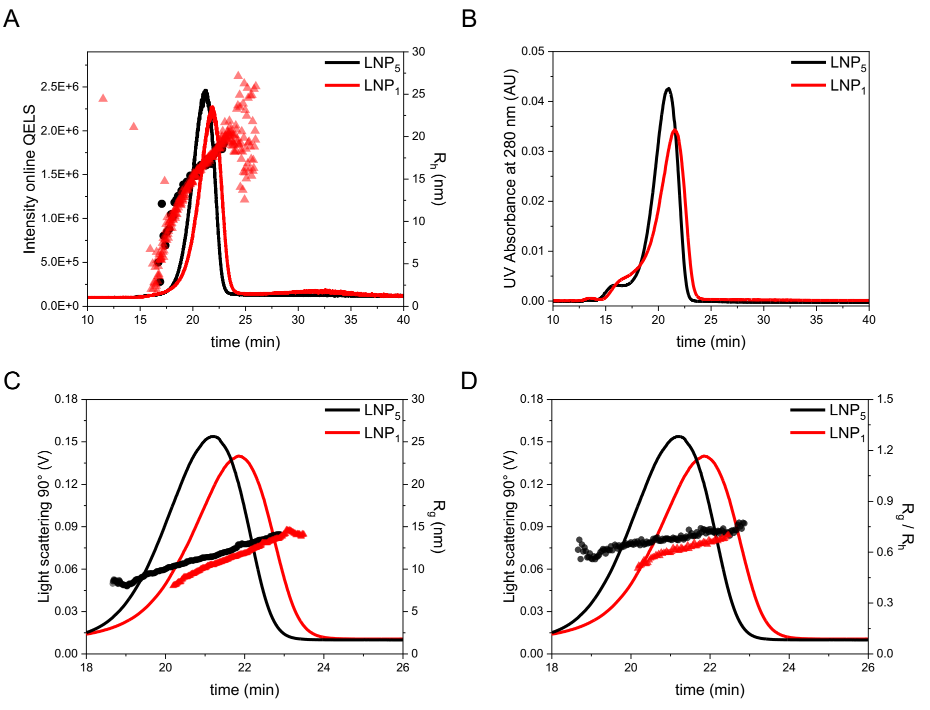

Figure S4: AF4 separation of unloaded LNP$_5$, 5.5% lipid fraction and unloaded LNP$_1$, 1.1% lipid fraction, illustrating the influence of the percentage lipid fraction on the size distribution. **(A)** Overlay of online QELS intensity vs. time, highlighting the R_h distribution across the eluting peak. **(B)** Overlay of UV absorbance at 280 nm vs. time. **(C)** Overlay of light scattering signal vs. time, highlighting the R_g distribution across the eluting peak. **(D)** Overlay of light scattering signal vs. elution time with R_g/R_h shape factor across the peak.

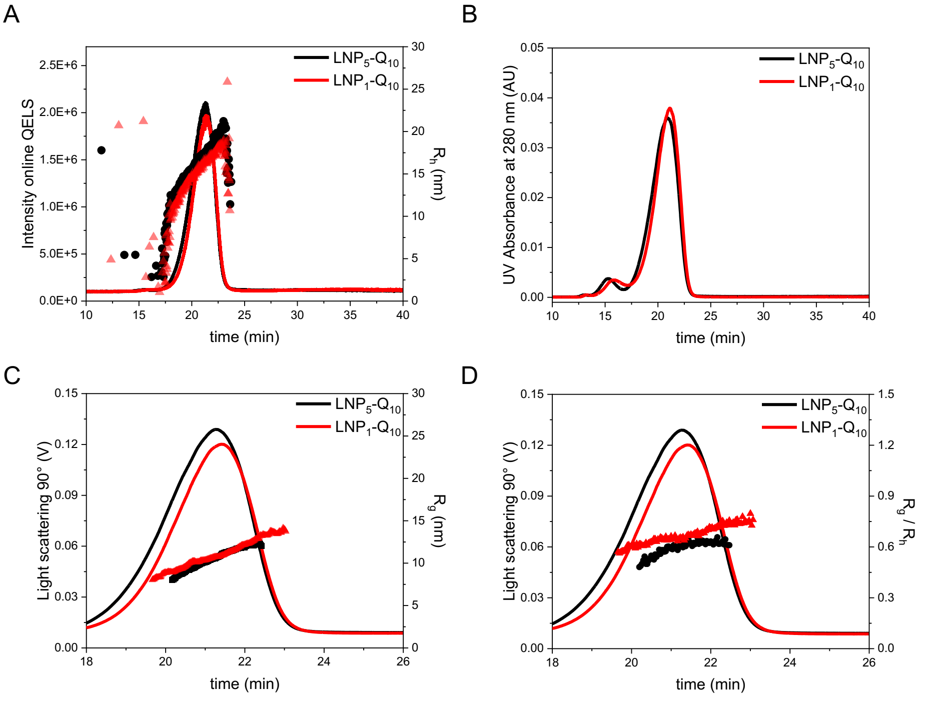

Figure S5: AF4 separation of LNP$_5$-Q$_{10}$, 5.5% lipid fraction with 10% quinine incorporation and LNP$_1$-Q$_{10}$, 1.1% lipid fraction with 10% quinine incorporation, to study the influence of the lipid fraction and percentage drug incorporation. **(A)** Overlay of online QELS intensity vs. time, highlighting the R_h distribution across the eluting peak. **(B)** Overlay of UV absorbance at 280 nm vs. time. **(C)** Overlay of light scattering signal vs. time, highlighting the R_g distribution across the eluting peak. **(D)** Overlay of light scattering signal vs. elution time with R_g/R_h shape factor across the peak.

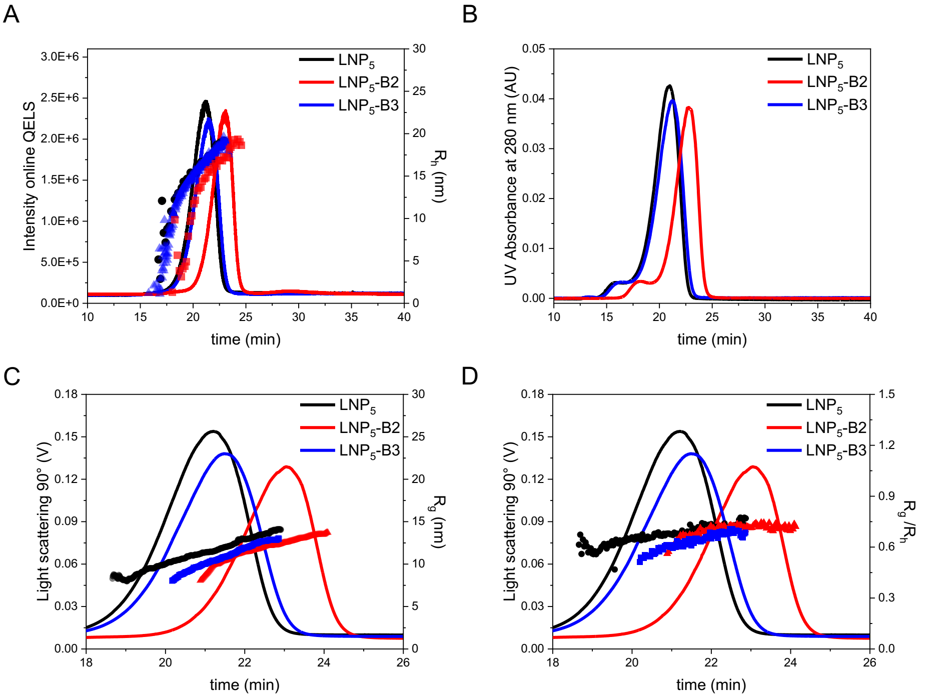

Figure S6: Batch-to-Batch comparison of the unloaded LNP5 with 5.5% lipid fraction formulations. **(A)** Overlay of online QELS intensity vs. time, highlighting the R_h distribution across the eluting peak. **(B)** Overlay of UV absorbance at 280 nm vs. time. **(C)** Overlay of light scattering signal vs. time, highlighting the R_g distribution across the eluting peak. **(D)** Overlay of light scattering signal vs. elution time with R_g/R_h shape factor across the peak.

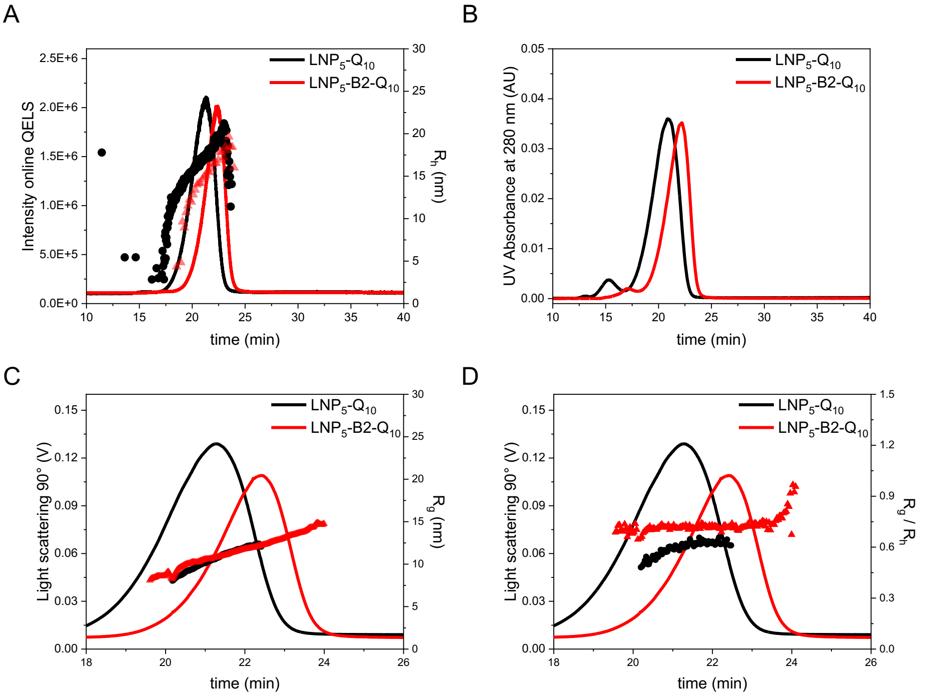

Figure S7: Batch-to-Batch comparison of the LNP5 with 10% quinine incorporation. **(A)** Overlay of online QELS intensity vs. time, highlighting the R_h distribution across the eluting peak. **(B)** Overlay of UV absorbance at 280 nm vs. time. **(C)** Overlay of light scattering signal vs. time, highlighting the R_g distribution across the eluting peak. **(D)** Overlay of light scattering signal vs. elution time with R_g/R_h shape factor across the peak.

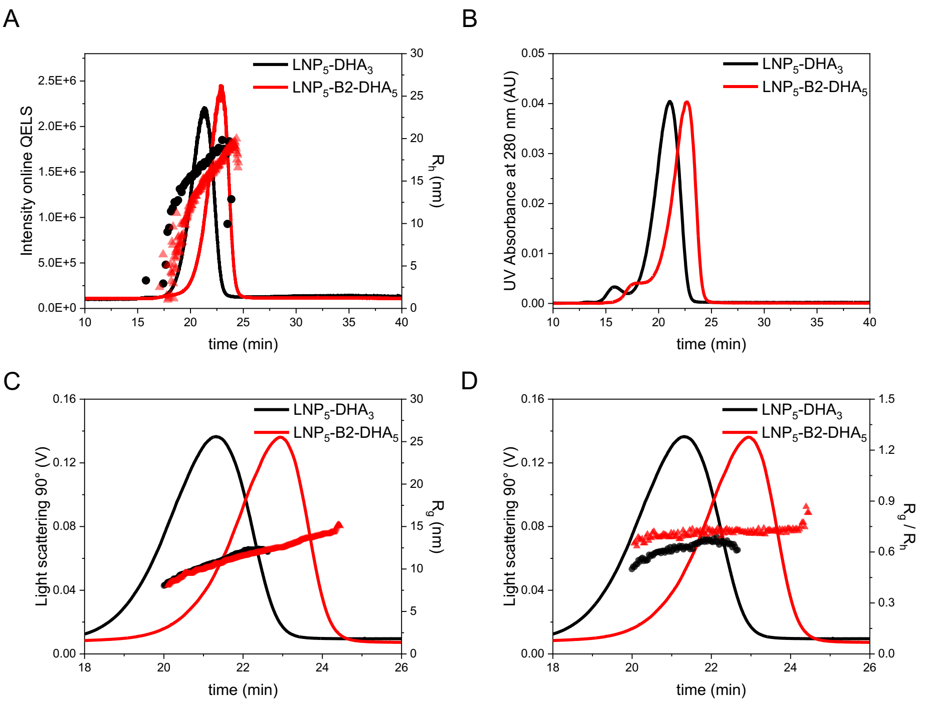

Figure S8: Comparison of LNP_5 with 3% and 5% dihydroartemisinin incorporation, respectively. **(A)** Overlay of online QELS intensity vs. time, highlighting the R_h distribution across the eluting peak. **(B)** Overlay of UV absorbance at 280 nm vs. time. **(C)** Overlay of light scattering signal vs. time, highlighting the R_g distribution across the eluting peak. **(D)** Overlay of light scattering signal vs. elution time with R_g/R_h shape factor across the peak.

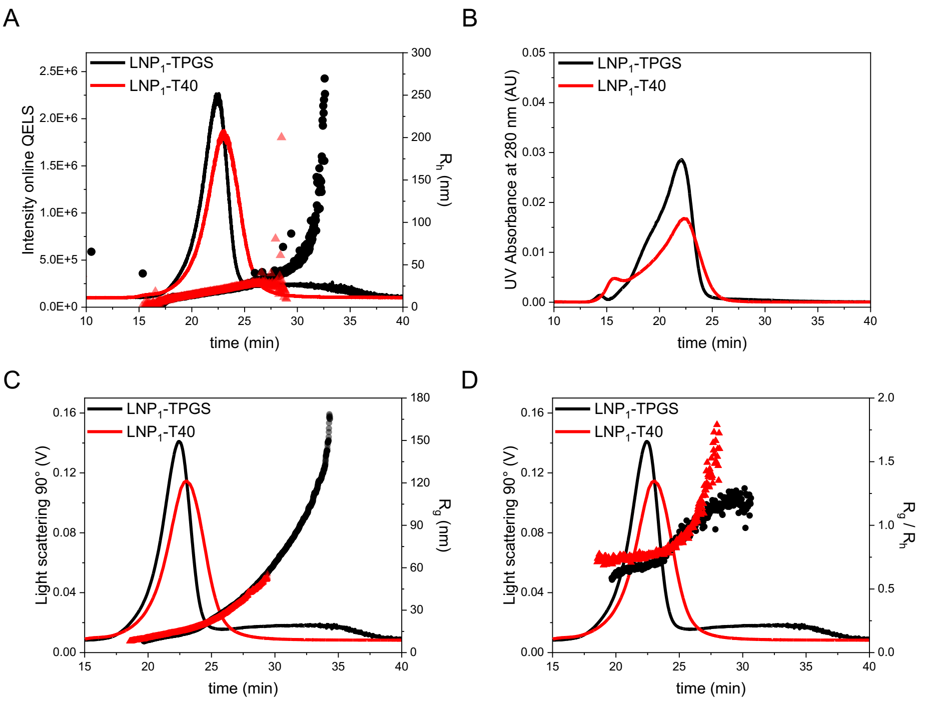

Figure S9: Comparison of AF4 separation of two LNP_1 samples with different surfactants. **(A)** Overlay of online QELS intensity vs. time, highlighting the R_h distribution across the eluting peak. **(B)** Overlay of UV absorbance at 280 nm vs. time. **(C)** Overlay of light scattering signal vs. time, highlighting the R_g distribution across the eluting peak. **(D)** Overlay of light scattering signal vs. elution time with R_g/R_h shape factor across the peak.

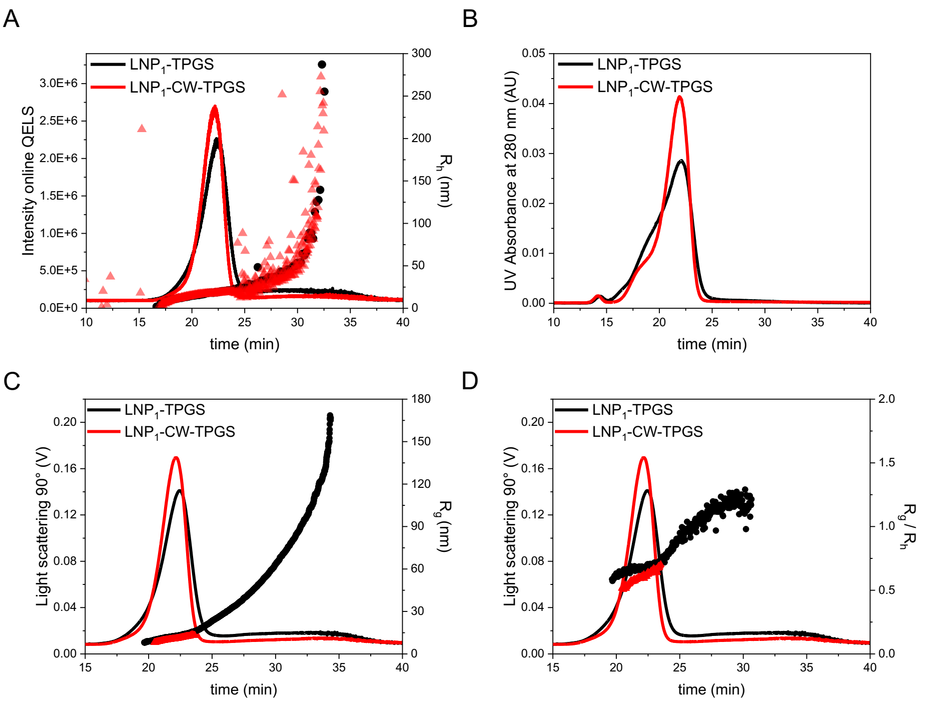

Figure S10: Comparison of AF4 separation of two LNP$_1$ samples with different lipid cores, but with the same surfactant (TPGS). LNP$_1$-TPGS has a lipid core that consists of carnauba wax and red palm oil, whereas LNP$_1$-CW-TPGS has a lipid core that consists of carnauba wax only. **(A)** Overlay of online QELS intensity vs. time, highlighting the R$_h$ distribution across the eluting peak. **(B)** Overlay of UV absorbance at 280 nm vs. time. **(C)** Overlay of light scattering signal vs. time, highlighting the R$_g$ distribution across the eluting peak. **(D)** Overlay of light scattering signal vs. elution time with R$_g$/R$_h$ shape factor across the peak.

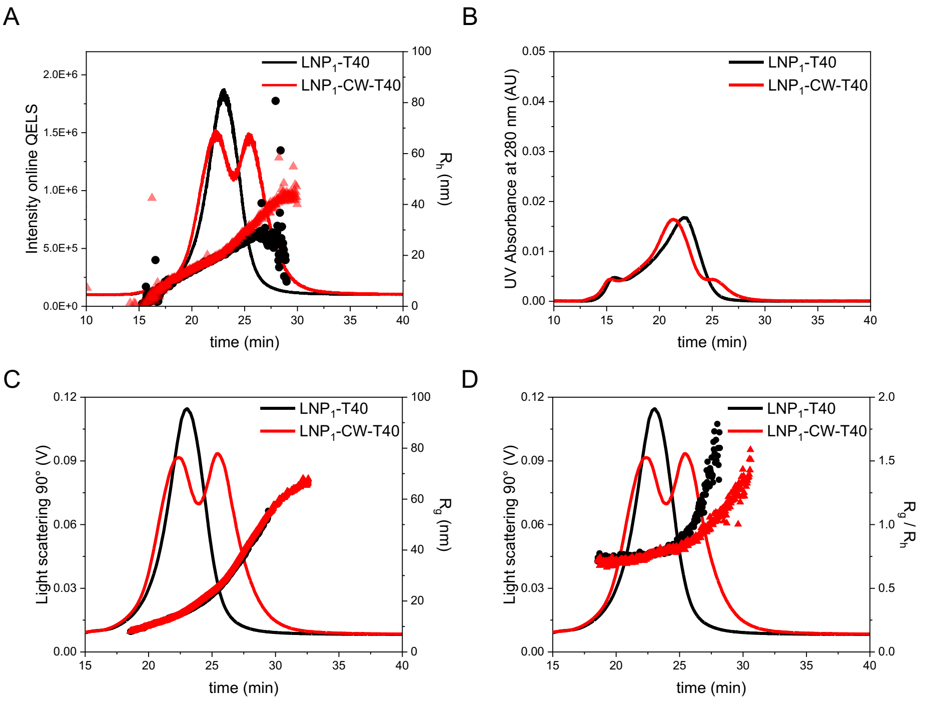

Figure S11: Comparison of AF4 separation of two LNP$_1$ samples with different lipid cores, but with similar surfactant (Polysorbate 40). LNP$_1$-T40 has a lipid core that consists of carnauba wax and red palm oil, whereas LNP$_1$-CW-T40 has a lipid core that consists of carnauba wax only. **(A)** Overlay of online QELS intensity vs. time, highlighting the R$_h$ distribution across the eluting peak. **(B)** Overlay of UV absorbance at 280 nm vs.

time. **(C)** Overlay of light scattering signal vs. time, highlighting the R_g distribution across the eluting peak. **(D)** Overlay of light scattering signal vs. elution time with R_g/R_h shape factor across the peak.

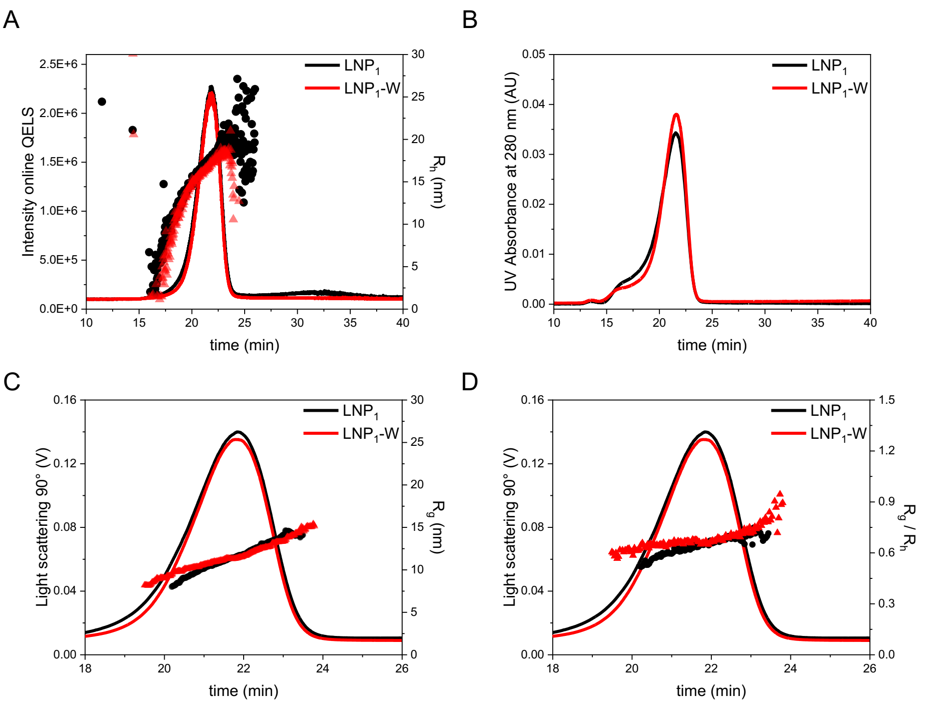

Figure S12: Comparison of AF4 separation of two LNP_1 formulations prepared in different dispersions. LNP_1 has been dispersed in 0.9 % (w/v) NaCl, and LNP_1-W has been dispersed in ultrapure water. **(A)** Overlay of online QELS intensity vs. time, highlighting the R_h distribution across the eluting peak. **(B)** Overlay of UV absorbance at 280 nm vs. time. **(C)** Overlay of light scattering signal vs. time, highlighting the R_g distribution across the eluting peak. **(D)** Overlay of light scattering signal vs. elution time with R_g/R_h shape factor across the peak.

Analytical validation and method performance

Table S6: Method validation via comparison of flow injection analysis (FIA) and full fractionation mode for representative LNP formulations

	UV peak area	% Recovery
**LNP_5**		
FIA (baseline)	0.1304	100
Focus FIA	0.1279	98.1
Full method	0.1246	95.5
**LNP_5-Q_{10}**		
FIA (baseline)	0.1591	100
Focus FIA	0.1337	84
Full method	0.1311	82

** All recovery calculations were performed in duplicate (n=2); injected volume 25 µL; c = 0.57 mg/mL.*

Table S7: Within-batch reproducibility of representative LNP formulations

Sample	Injection number	Retention time of main peak at peak height of UV (min)	Rh at peak maximum (nm)	Peak area (UV 280 nm)
**LNP_5**	1	20.92	16.4 ± 0.4	0.1261
	2	20.96	17.1 ± 0.4	0.1274
	3	21.05	16.9 ± 0.4	0.1284
Retention time RSD %	0.32			
Rh RSD %	2.15			
Area RSD %	0.91			
**LNP_5-Q_{10}**	1	20.98	17.5 ± 0.4	0.1140
	2	20.94	17.7 ± 0.4	0.1143
	3	20.92	17.2 ± 0.4	0.1144
Retention time RSD %	0.15			
R_h RSD %	1.44			
Area RSD %	0.18			
**LNP_5-DHA_3**	1	21.09	17.3 ± 0.4	0.1089
	2	21.08	174 ± 0.4	0.1134
	3	21.08	17.1 ± 0.4	0.1148
Retention time RSD %	0.03			
R_h RSD %	0.88			
Area RSD %	2.75			

Table S8: Batch-to-batch consistency of hydrodynamic radius (R_h) and radius of gyration (R_g) across independent LNP formulation

Batch	Mean Rh at peak maximum (nm)	Rh RSD %	Mean Rg at peak maximum (nm)	Rg RSD %
**LNP_5**	16.8	2.15	11.4	0.88
**LNP_5-B2**	17.3	0.79	12.6	1.21
**LNP_5-B3**	17.3	2.22	10.8	0.93
Batch-to-batch R_g RSD%	8.05			
Batch-to-batch R_h RSD%	1.75			
**LNP_5-Q_{10}**	17.5	1.44	10.4	1.46
**LNP_5-B2-Q_{10}**	16.8	1.53	12.2	0.47
**Batch-to-batch R_g RSD%**	11.23			
**Batch-to-batch R_h RSD%**	2.80			

Within-batch precision was the RSD < 5%. Between batches, R_h remained consistent (RSD < 3%), while R_g varied by 8-11%, corresponding to a shape factor of 0.60-0.73, thus providing structural insight into internal mass distributions.

Table S9: Summary of Radius of gyration (R_g), Hydrodynamic radius (R_h) and R_g/R_h ratio for various LNP formulations and batches

Sample	R_g (a)	R_h (a)	$\frac{R_g}{R_h}$ (a)
LNP_5	11.4 ± 0.3	16.8 ± 0.4	0.68
LNP_5-Q_{10}	10.4 ± 0.5	17.4 ± 0.4	0.60
LNP_5-DHA_3	10.6 ± 0.3	17.3 ± 0.4	0.61
LNP_1	11.6 ± 0.3	17.9 ± 0.4	0.65
LNP_1-Q_{10}	10.8 ± 0.3	16.4 ± 0.4	0.66
LNP_1-W	11.2 ± 0.3	17.3 ± 0.4	0.64
LNP_1-TPGS	12.9 ± 0.3	18.8 ± 0.4	0.69
LNP_1-T40	16.0 ± 0.2	20.5 ± 0.5	0.78
LNP_1-CW-TPGS	10.9 ± 0.3	17.5 ± 0.4	0.62
LNP_1-CW-T40 (first eluting peak)	14.7 ± 0.3	19.5 ± 0.4	0.75
LNP_1-CW-T40 (second eluting peak)	25.5 ± 0.2	30.5 ± 0.7	0.84
LNP_5-B2	12.6 ± 0.4	17.3 ± 0.4	0.73
LNP_5-B2-Q_{10}	12.2 ± 0.3	16.8 ± 0.4	0.73
LNP_5-B2-DHA_5	12.4 ± 0.3	17.2 ± 0.4	0.72
LNP_5-B3	10.8 ± 0.3	17.3 ± 0.4	0.62

(a) Determined at peak height

Analytical method for quinine quantification

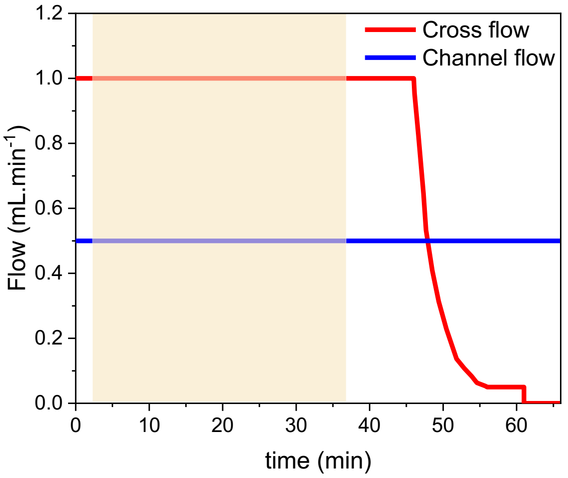

Figure S13: AF4 flow profile with the cross-flow coupled to the UV-Vis.

Table S10: Experimental conditions for the characterization of LNP by AF4-MD

**Conditions**	
Injection volume:	25 µL
Spacer:	350 µm
Membrane:	10 kDa (regenerate cellulose, Wyatt)
Detector flow:	0.3 mL min^{-1}
Eluent:	10 mM PBS buffer at pH 7.4, containing 200 mg L^{-1} NaN_3
Channel:	Eclipse short channel with DCM, Wyatt Technologies Corp.
**Detector**	
Agilent 1260 Infinity II MWD	Wavelength: 250 nm
Multi-angle light scattering (MALS) with online QELS (DAWN Neon MALLS, Wyatt Technologies Corp.)	18 angles, operating at a wavelength of 659 nm
OptiLab dRI	Operating at a wavelength of 658 nm
Method:	Isocratic step with a V_x of 1 mL min^{-1} for 46 min (Elution; Focus; Focus Inject; Focus, Elution).<br>Exponential V_x gradient from 1 to 0.05 mL min^{-1} within 10 min was used.<br>Isocratic V_x of 0.05 mL min^{-1} for 5 min.<br>Isocratic V_x of 0 mL min^{-1} for 5 min.<br>Injection flow rate: 0.2 mL min^{-1}

Table S11: UV detector calibration data at 250nm and sensitivity metrics

Quinine concentration ($mg.mL^{-1}$)	Mean peak area at 250 nm[a]	Standard deviation of area
0.0519	1.19×10^{-2}	1.77×10^{-4}
0.0636	1.78×10^{-2}	6.92×10^{-4}
0.0865	2.64×10^{-2}	1.27×10^{-4}
0.106	3.19×10^{-2}	6.68×10^{-4}
0.121	3.84×10^{-2}	7.42×10^{-4}
0.1484	4.68×10^{-2}	3.92×10^{-4}
0.1730	5.73×10^{-2}	6.35×10^{-4}
0.2120	7.00×10^{-2}	7.15×10^{-4}
**Average calibration σ**		5.45×10^{-4}
**Regression**		
Slope S	0.3586	
Intercept	-0.0056	
R^2	0.9983	
**LOD = 3.3σ /S**	0.0050 mg mL^{-1}	
**LOQ = 10σ /S**	0.0152 mg mL^{-1}	

a) n =3

The sensitivity of the UV detector (250 nm) was assessed by determining the limit of detection and the limit of quantification. These were calculated from the slope (S= 0.3586) and the average standard deviation of the response ($\sigma = 5.45 \times 10^{-4}$). The obtained limits indicate that the method is suitable for quantifying low-concentration drug concentrations, where the cross-flow outlet is coupled to the UV-Vis detector.

Cryo-TEM characterization of LNPs

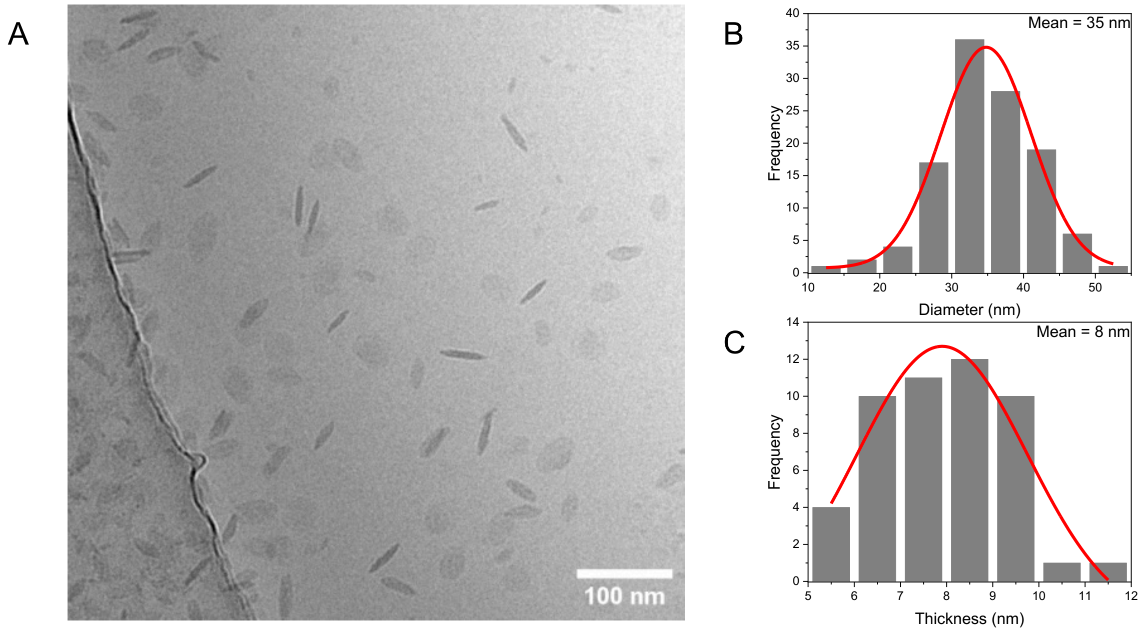

Figure S14: Cryo-TEM analysis of LNP$_5$. **(A)** Cryo-TEM image of the particles. **(B)** Particle diameter distribution measured from 20-40 particles across three images, fitted with a Gaussian distribution. **(C)** Particle thickness distribution measured from 10-20 particles across three images, fitted with a Gaussian distribution.

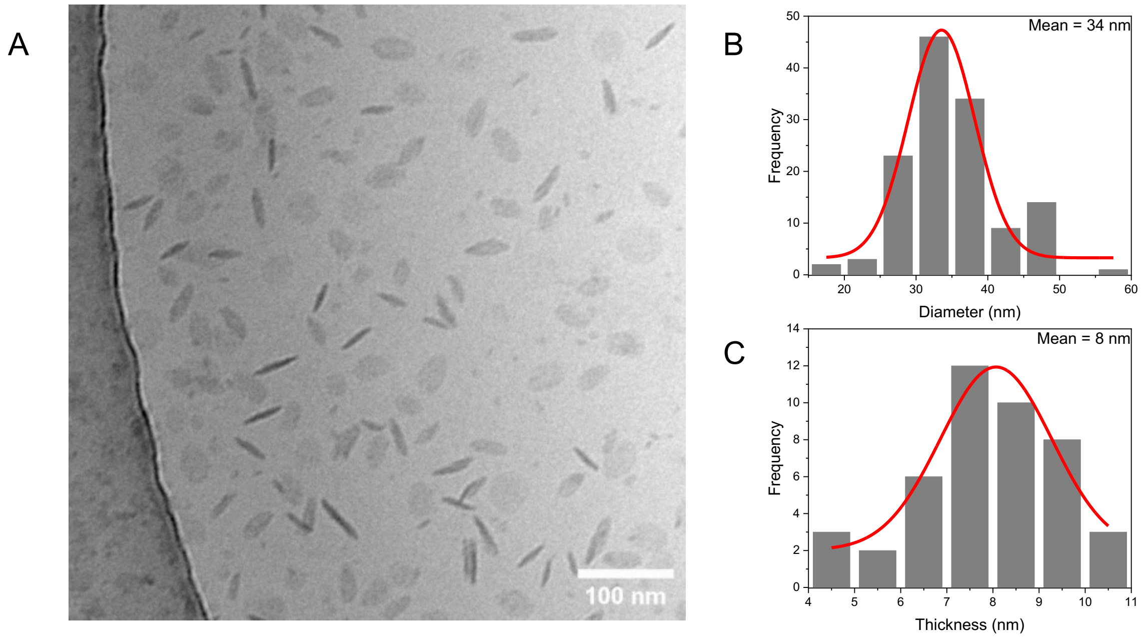

Figure S15: Cryo-TEM analysis of LNP$_5$-Q$_{10}$. **(A)** Cryo-TEM image of the particles. **(B)** Particle diameter distribution measured from 20-40 particles across three images, fitted with a Gaussian distribution. **(C)** Particle thickness distribution measured from 10-20 particles across three images, fitted with a Gaussian distribution.

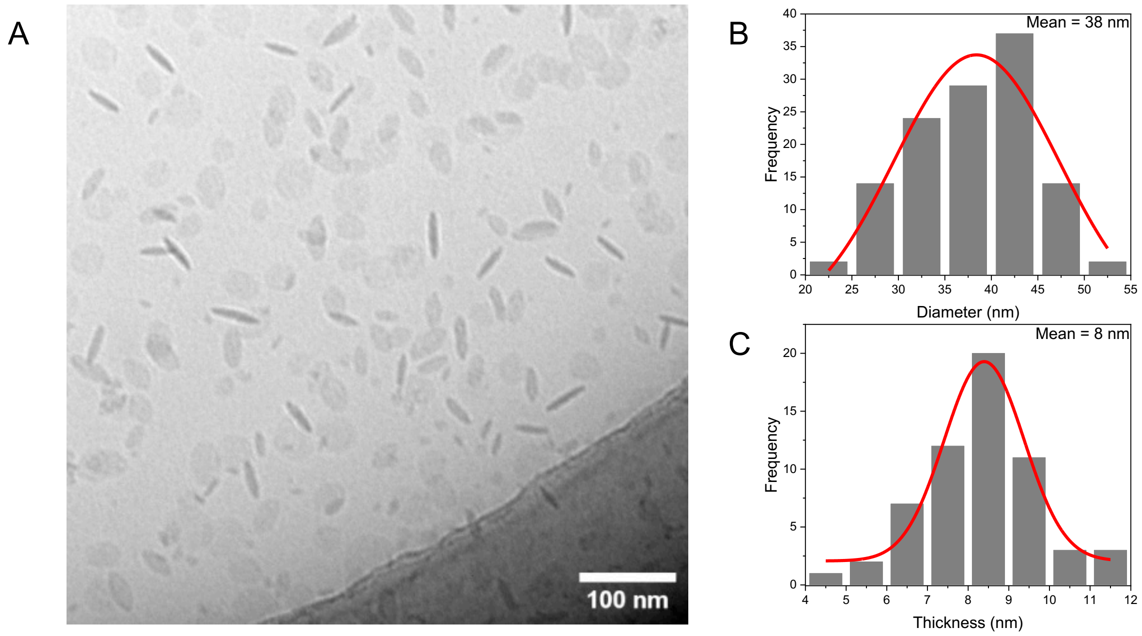

Figure S16: Cryo-TEM analysis of LNP_5-DHA_3. **(A)** Cryo-TEM image of the particles. **(B)** Particle diameter distribution measured from 20-40 particles across three images, fitted with a Gaussian distribution. **(C)** Particle thickness distribution measured from 10-20 particles across three images, fitted with a Gaussian distribution.

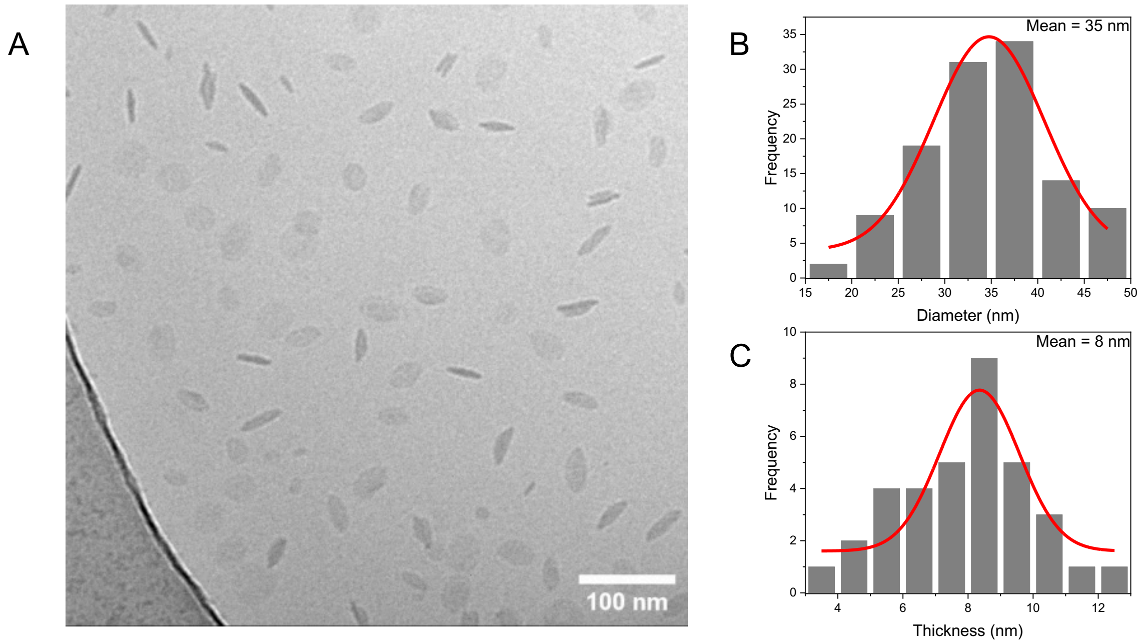

Figure S17: Cryo-TEM analysis of LNP_1-W. **(A)** Cryo-TEM image of the particles. **(B)** Particle diameter distribution measured from 20-40 particles across three images, fitted with a Gaussian distribution. **(C)** Particle thickness distribution measured from 10-20 particles across three images, fitted with a Gaussian distribution.

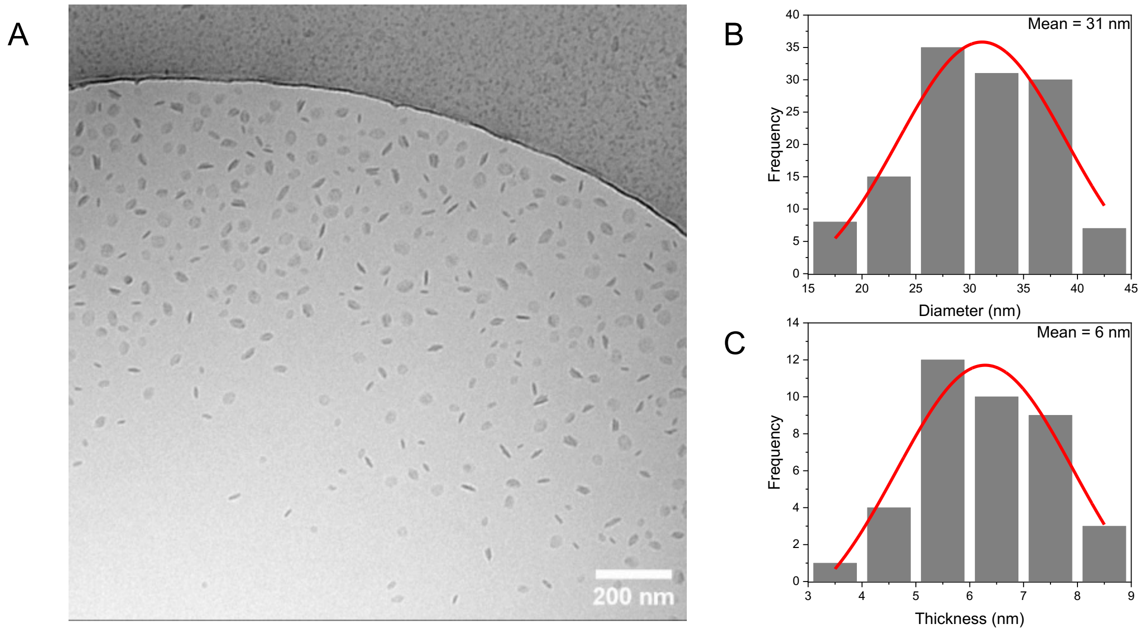

Figure S18: Cryo-TEM analysis of LNP_1-CW-TPGS. **(A)** Cryo-TEM image of the particles. **(B)** Particle diameter distribution measured from 20-40 particles across three images, fitted with a Gaussian distribution. **(C)** Particle thickness distribution measured from 10-20 particles across three images, fitted with a Gaussian distribution.

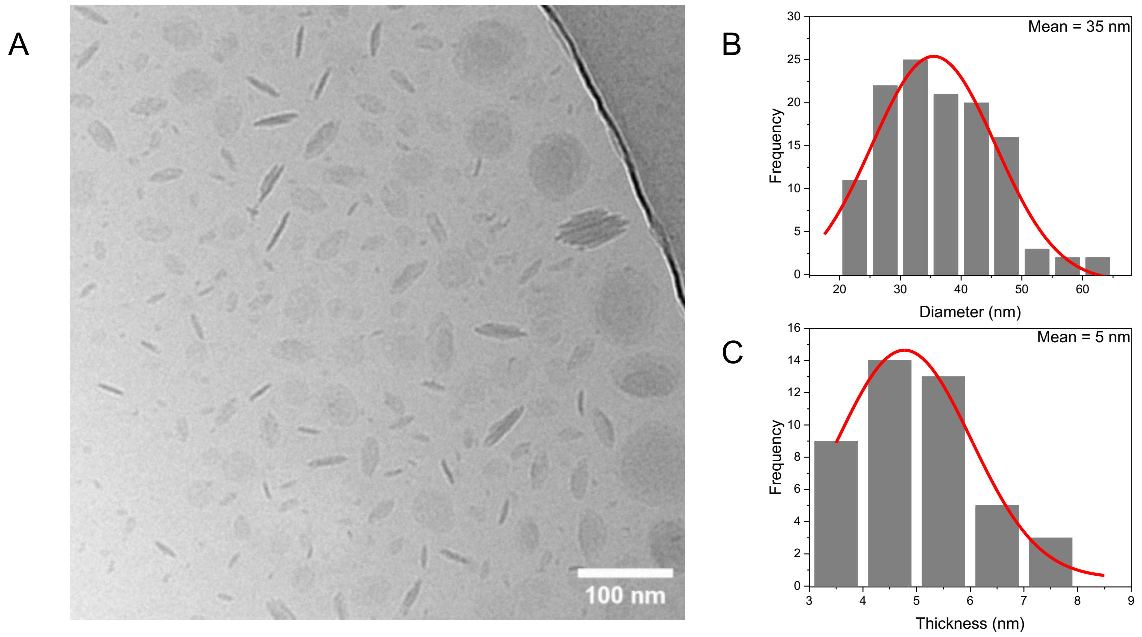

Figure S19: Cryo-TEM analysis of LNP_1-CW-T40. **(A)** Cryo-TEM image of the particles. **(B)** Particle diameter distribution measured from 20-40 particles across three images, fitted with a Gaussian distribution. **(C)** Particle thickness distribution measured from 10-20 particles across three images, fitted with a Gaussian distribution.

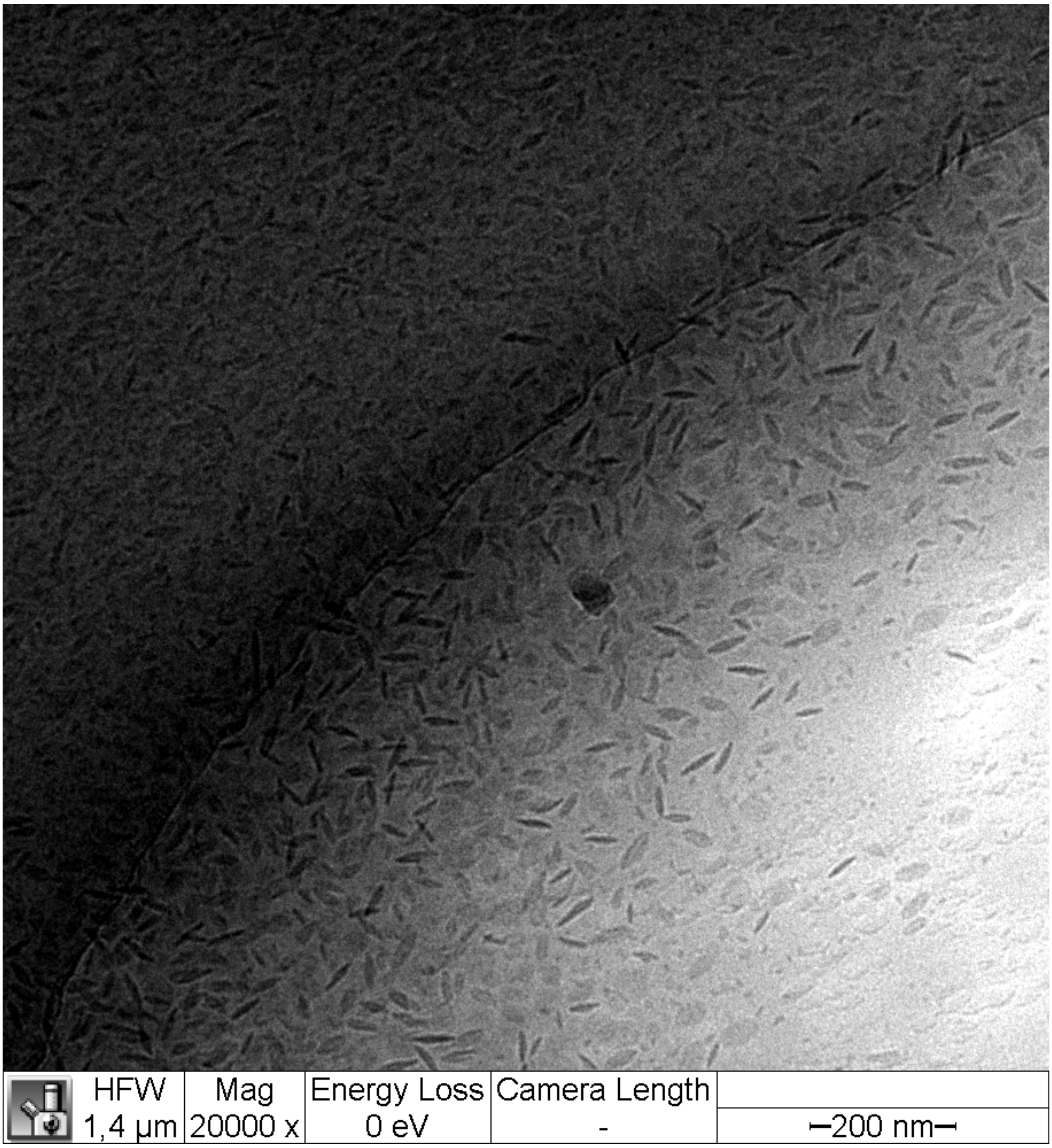

Figure S20: A three-dimensional rendering of the cryo-TEM micrograph of the LNPs. A video is provided as Supplementary Video 1.

Characterization of LNP using SAXS

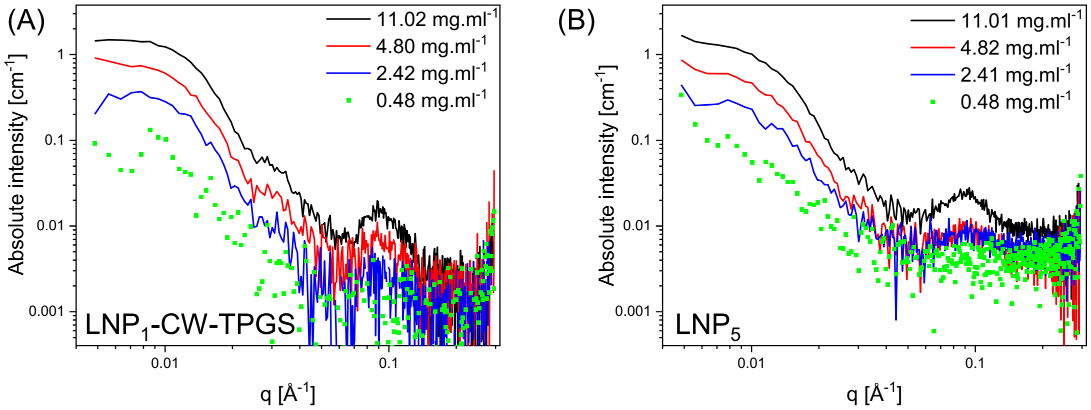

Figure S21: SAXS data converted to absolute intensity for LNP1-CW-TPGS (A) and LNP5 (B) to evaluate R_g for four different concentrations of LNP1-CW-TPGS and LNP5 in ultrapure water.

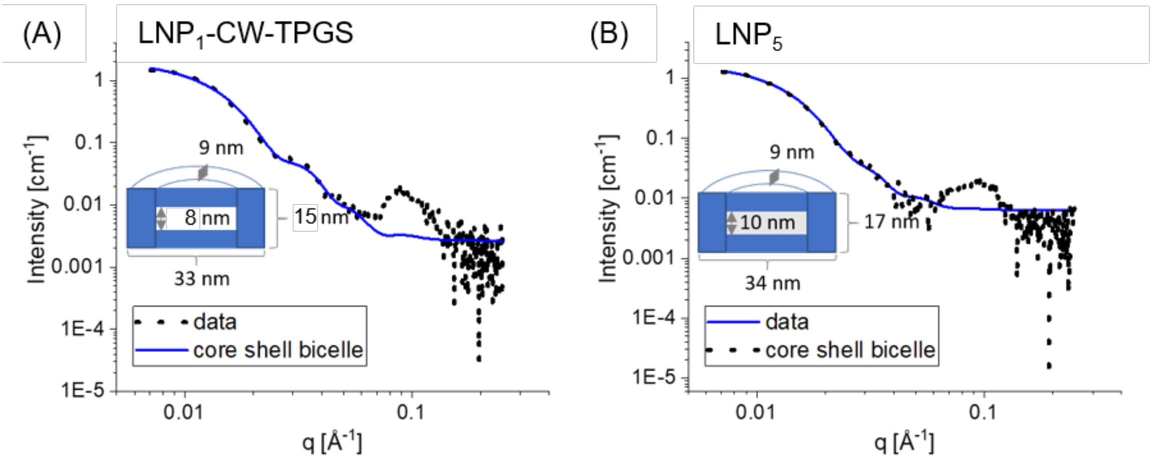

Figure S22: SAXS data and modelling by a core-shell bicelle for LNP1-CW-TPGS (A) and LNP5 (B) in water at a concentration of 11.02 mg mL^{-1} based on lipid fraction. The reflection at q = 0.09 Å^{-1} cannot be captured by the model. Sketches of the modelled shape, considering two different SLDs of the core and the shell of the bicelle, are added to the graphs. For the LNP1-CW-TPGS, which consists only of carnauba wax and TPGS, the SLD of the core was close to that of the solvent water.

Characterization of LNP loading using fluorescence spectroscopy

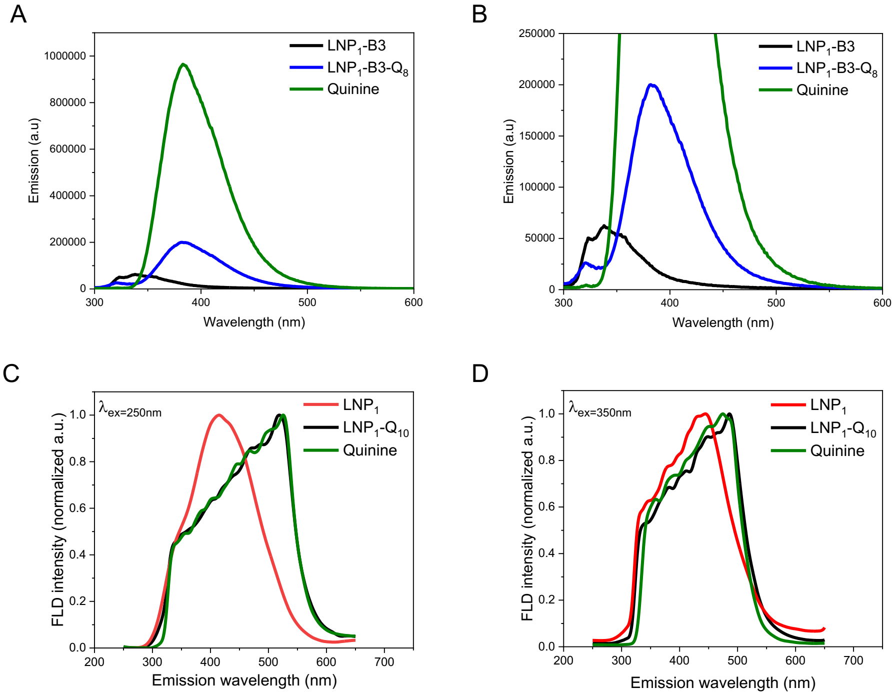

Figure S23: **(A)** Fluorescence emission spectra of drug-free LNP1-B3 and LNP1-B3-Q8 loaded with quinine recorded at an excitation wavelength of 288 nm show an overlap between quinine and quinine-loaded LNP. **(B)** Enlarged fluorescence spectra. Measurements were conducted at room temperature using a slit width of 3 nm. **(C)** and **(D)** Fluorescence detection of the LNPs shows that the quinine is located in the particle due to increased intensity of the fluorescence signal at the same elution volume as the particle. The concentration of the particles is kept the same; thus, the increasing absorption intensity and broader absorption wavelength after excitation at 250 and 350 nm are the result of the quinine in the loaded LNP1-Q10 compared to the pure LNP1.

Table S12: Summarizes the nanoparticle synthesis process and formulation parameters

Step	Stage/Sub-step	Description	Key Parameters/Conditions	Notes/Implications
1	Lipid Phase Preparation	Mix lipids + surfactants; heat until clear melt	Lipids: carnauba wax + red palm oil/tocotrienols (~1:1) Surfactants: TPGS + polysorbate 40 Heat: 90°C ±2°C, stir 500 rpm	Core phase; ensures homogeneous melt for API incorporation
2	Lipid Phase Preparation (cont.)	Add lipophilic API; stir until fully dissolved	Stir at 90°C until clear	Critical for high encapsulation efficiency (>90%); API must be lipophilic
3	Aqueous Phase Preparation (Parallel to Steps 1-2)	Prepare deionized water + NaCl; heat	NaCl for isotonicity Heat: 90°C ±2°C	Performed in parallel for efficiency; timing must align with lipid phase readiness
4	Emulsification	Add hot aqueous phase dropwise to hot lipid phase with stirring	Continuous stirring → Initial W/O emulsion	Dropwise addition prevents premature inversion
5	Phase Inversion	Cool gradually with reduced stirring	Cool to 20°C ±2°C Reduced stirring: 300 rpm → Phase inversion & SLN self-assembly	Thermodynamic driver; yields 20-40 nm particles, narrow PDI
6	Finalization	Form CellInject SLN dispersion	20-40 nm particles, narrow PDI, high encapsulation efficiency (EE)	Ready dispersion; "in-cell only" release mechanism enabled
7a	Purification<br>(Optional Branch - Yes)	Dialysis to remove residuals	50 kDa MWCO tubing vs 0.9% NaCl 48 hours, refresh medium every 12 hours Until no residual surfactant/drug	Recommended for clinical-grade; ensures purity, stability
7b	Finalization (Optional Branch - No)	Ready-to-use particles	Store refrigerated	For research/lab use

Bioactivity and biocompatibility studies

Table S13: Human primer

**Gene**	**Accession number**	**Forward primer**<br>**Reverse primer**
COL1a1	NM_000088.4	**F:** GTCGCACTGGTGATGCTG<br>**R:** GGTGGTGTCCACCTCGAG
COL3a1	NM_000090.4	**F:** CCATTGCTGGGATTGGAG<br>**R:** GTCCACCAGTGTTTCCGTG
EDA-FN	NM_002026.2	**F:** CCAGTCCACAGCTATTCCTG<br>**R:** ACAACCACGGATGAGCTG
ACTA2 (aSMA)	NM_001141945.3	**F:** AGACCCTGTTCCAGCCATC<br>**R:** TGCTAGGGCCGTGATCTC
TNF	NM_000594.3	**F:** GAGTGACAAGCCTGTAGCCCATGTTGTAGCA<br>**R:** GCAATGATCCCAAAGTAGACCTGCCCAGACT
IL-1b	NM_000576.3	**F:** GACACATGGGATAACGAGGC<br>**R:** ACGCAGGACAGGTACAGATT
MCP1	NM_002982.4	**F:** CAGCCAGATGCAATCAATGC<br>**R:** GTCTTCGGAGTTTGGGTTTGC
IL-10	NM_000572.3	**F:** GATCTCCGAGATGCCTTCAG<br>**R:** CATGCGCCTTGATGTCTG
PPARγ	NM_138712.5	**F:** AGTCCTCACAGCTGTTTGCCAAGC<br>**R:** GAGCGGGTGAAGACTCATGTCTGTC
ADIPOQ	NM_001177800.2	**F:** ACCCAGAGCTGTGGACTTTG<br>**R:** GTAGTCCTTCCAAGACAGGACG
PNPLA2 (ATGL)	NM_020376.4	**F:** GAGTGACATCTGTCCGCAGG<br>**R:** GCGAGTAATCCTCCGCTTGG
RPS26	NM_001029	**F:** CAATGGTCGTGCCAAAAAG<br>**R:** TTCACATACAGCTTGGGAAGC

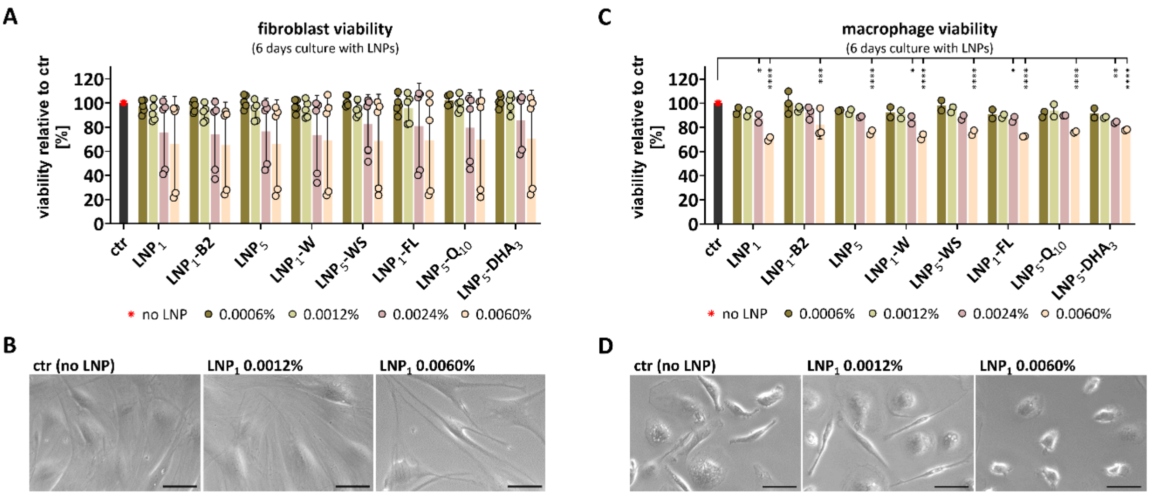

Figure S24: Cell viability after culture with LNP. Human dermal fibroblasts and monocyte-derived human macrophages were cultured for 6 days with different LNPs at different concentrations. **(A)** Viability of fibroblasts was determined with the XTT assay. n=4 different fibroblast donors. **(B)** Microscopic evaluation of fibroblasts. Scale = 50 µm. **(C)** Viability of macrophages was determined with the XTT assay. n=3 different macrophage donors. **(D)** Microscopic evaluation of macrophages. Scale = 50 µm. A/C) Two-way ANOVA with Tukey‘s multiple comparisons test. Significant differences compared to ctr (no LNP) are indicated. * $p<0.05$, *** $p<0.001$, **** $p<0.0001$. LNP concentrations are indicated as % lipid fraction. LNP_1, LPN_1-B2, LNP_5 comprise different batches of LNP constituted in 0.9% NaCl at stock concentrations of 1.1%, 1.2% and 5.5% lipid fraction, respectively. LNP_1-W are constituted in pure water. LNP_5-WS, the residual surfactants were removed. LNP_1-FL, LNP_5-Q_{10}, and LNP_5-DHA_3 comprise batches of LNP_1 loaded with fluorescein (FL) for LNP tracking or with the drugs quinine (Q) or dihydroartemisinin (DHA).

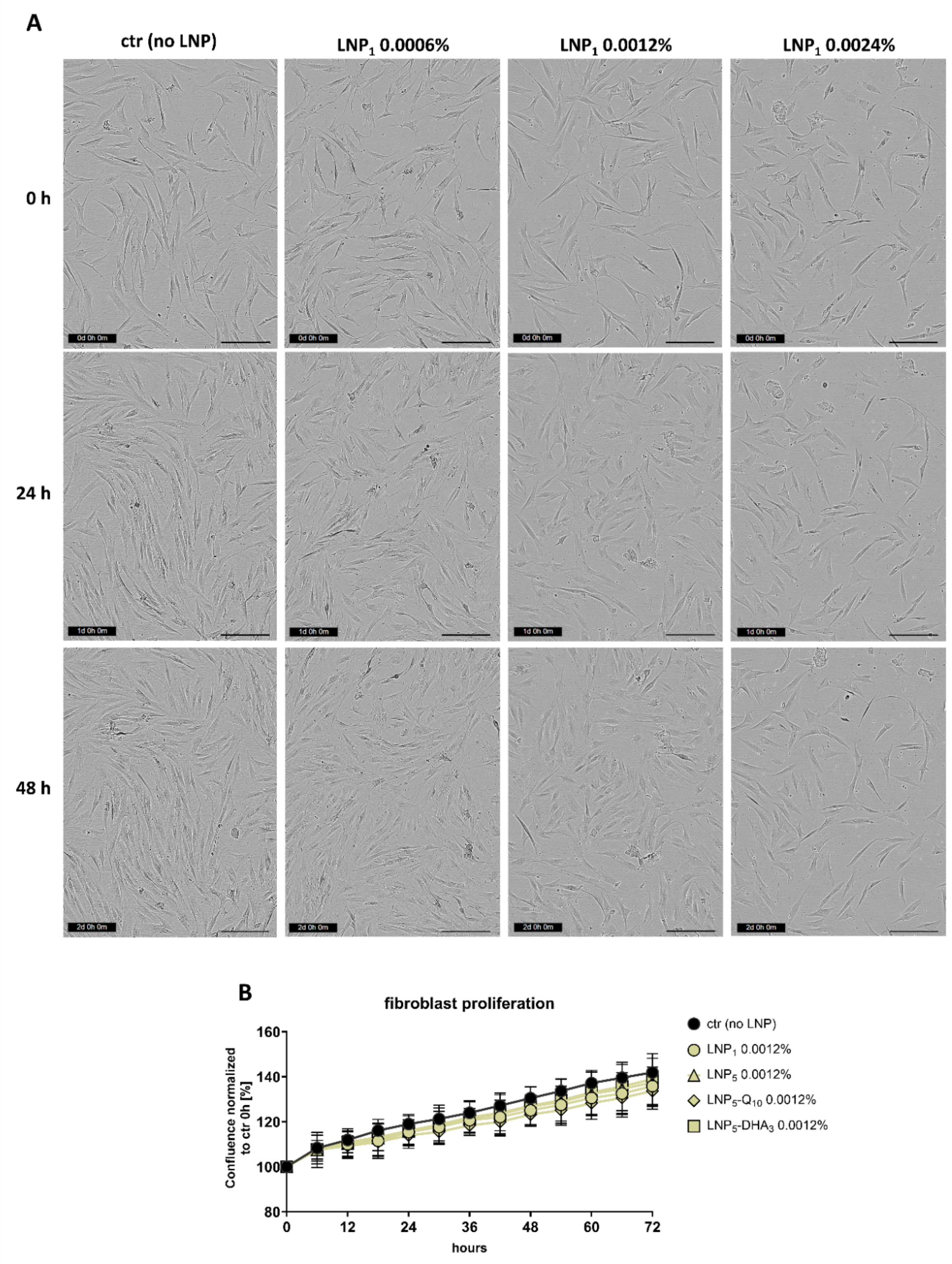

Figure S25: Proliferation of fibroblasts monitored via IncuCyte® live cell imaging. Human dermal fibroblasts were incubated with various LNP at different concentrations in the IncuCyte® S3. For a total of 72 h, 12 defined areas in each well with fibroblasts were photographed at a time interval of 6 h and used to determine fibroblast proliferation over 72 h. **(A)** Images of one representative area for the conditions indicated, photographed at 0 hours, 24 hours and 48 hours are shown (refers to **Figure 5A**). Scale = 200 µm. **(B)** Quantification of fibroblast proliferation with different LNP. n=4. Two-way ANOVA with Dunnett's multiple comparisons test was performed. No significant differences were observed. LNP concentrations are indicated as % lipid fraction. LNP_1, LNP_5, LNP_5-Q_{10}, and LNP_5-DHA_3 comprise different batches of LNP as outlined in **Figure 4**.

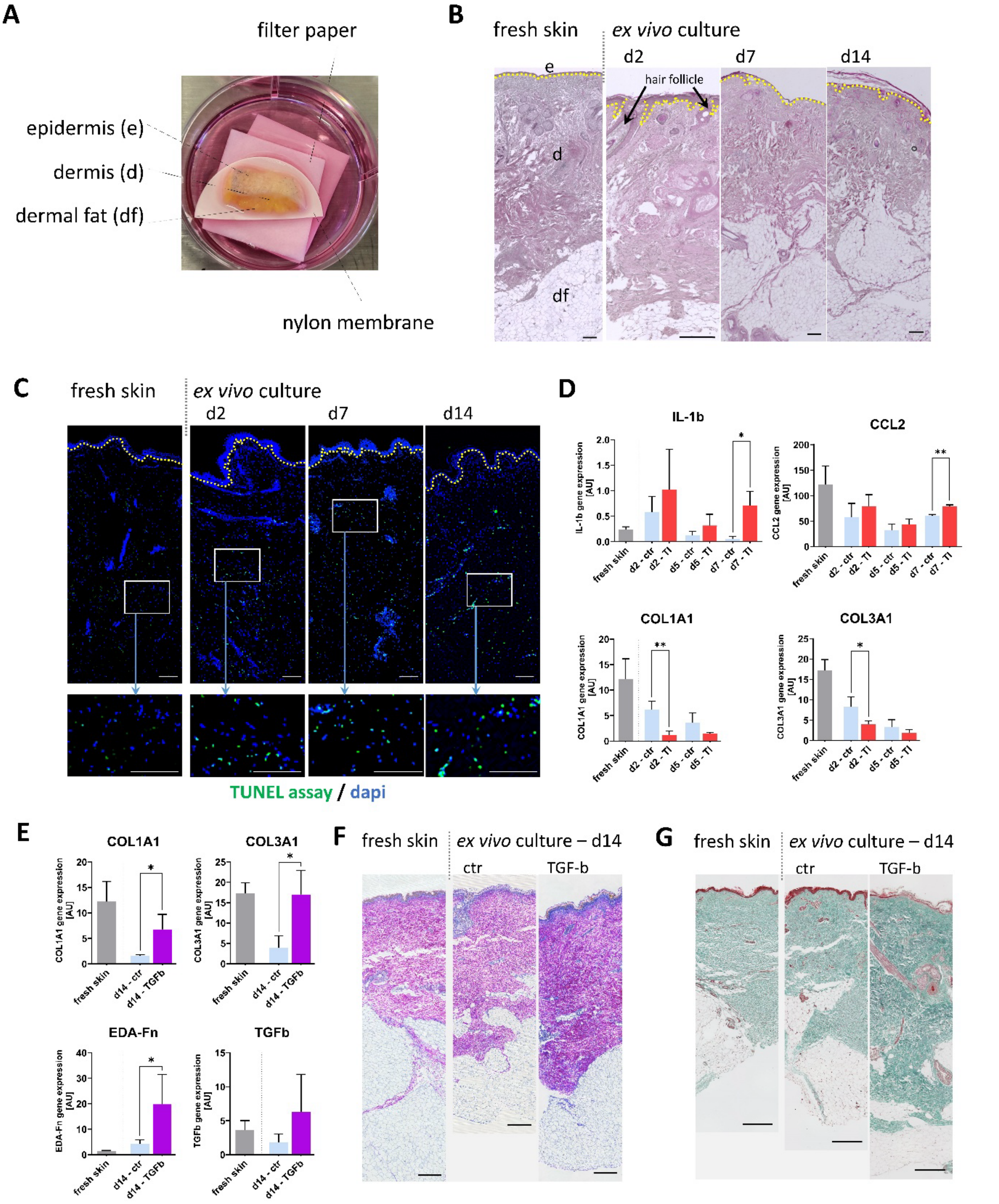

Figure S26: *Ex vivo* skin culture model. **A)** Image of human skin prepared for *ex vivo* culture in a culture plate on two filter papers and a nylon membran to ensure adequate medium and nutrient supply. **B)** Hematoxylin and Eosin (H&E) staining of histology section of fresh human skin and human skin cultured *ex vivo* for two days, 7 days and 14 days. Scale = 300µm. **C)** Evaluation of skin viability via TUNEL staining of histology sections of fresh human skin and human skin cultured *ex vivo* for two days, 7 days and 14 days. Scale = 200µm. **D)** Gene expression analysis of fresh human skin and human skin cultured *ex vivo* for two days, and 5 days that were stimulated with TNF/IL-1b (TI) or left unstimulated (ctr). The upregulation of pro-

inflammatory genes accompanied by reduced ECM gene expression demonstrates the functional responsiveness of the skin model to inflammatory stimuli. n=3, unpaired t-test: * $p < 0.05$; ** $p < 0.001$. **E)** Gene expression analysis of fresh human skin and human skin cultured *ex vivo* for 14 days that were stimulated with TGFb or left unstimulated (ctr). n=3, unpaired t-test: * $p < 0.05$. **F/G)** Herovici staining (F) and Masson's trichrome staining (G) detecting for collagen deposition in fresh human skin and in human skin cultured *ex vivo* for 14 days that were stimulated with TGFb or left unstimulated (ctr). Scale = 500µm. Increased ECM gene expression and deposition demonstrates the functional responsiveness of the skin model to pro-fibrotic stimuli.
